# Tandem repeats in giant archaeal Borg elements undergo rapid evolution and create new intrinsically disordered regions in proteins

**DOI:** 10.1371/journal.pbio.3001980

**Published:** 2023-01-26

**Authors:** Marie Charlotte Schoelmerich, Rohan Sachdeva, Jacob West-Roberts, Lucas Waldburger, Jillian F. Banfield

**Affiliations:** 1 Innovative Genomics Institute, University of California, Berkeley, California, United States of America; 2 Earth and Planetary Science, University of California, Berkeley, California, United States of America; 3 Bioengineering, University of California, Berkeley, California, United States of America; 4 Environmental Science, Policy and Management, University of California, Berkeley, California, United States of America; 5 Lawrence Berkeley National Laboratory, Berkeley, California, United States of America; Fred Hutchinson Cancer Research Center, UNITED STATES

## Abstract

Borgs are huge, linear extrachromosomal elements associated with anaerobic methane-oxidizing archaea. Striking features of Borg genomes are pervasive tandem direct repeat (TR) regions. Here, we present six new Borg genomes and investigate the characteristics of TRs in all ten complete Borg genomes. We find that TR regions are rapidly evolving, recently formed, arise independently, and are virtually absent in host *Methanoperedens* genomes. Flanking partial repeats and A-enriched character constrain the TR formation mechanism. TRs can be in intergenic regions, where they might serve as regulatory RNAs, or in open reading frames (ORFs). TRs in ORFs are under very strong selective pressure, leading to perfect amino acid TRs (aaTRs) that are commonly intrinsically disordered regions. Proteins with aaTRs are often extracellular or membrane proteins, and functionally similar or homologous proteins often have aaTRs composed of the same amino acids. We propose that Borg aaTR-proteins functionally diversify *Methanoperedens* and all TRs are crucial for specific Borg–host associations and possibly cospeciation.

## Abstract 2

This study shows that extrachromosomal elements associated with methane-consuming Methanoperedens archaea (known as Borgs) are scattered with nucleotide tandem repeats that evolve rapidly. When occurring within genes, the resulting amino acid repeats create largely intrinsically disordered regions.

## 1. Introduction

Metagenomics has led to the increasing discovery of microbial extrachromosomal elements (ECEs) from environmental samples [1,2]. This bears great importance, as it allows us to better understand evolutionary processes and functional roles of ECEs in natural systems and potentially use the ECEs, or elements of them, for genetic engineering. Using genome-resolved metagenomics, we recently discovered Borgs, which are unusually large ECEs. Based on gene content and co-occurrence patterns, Borgs associate with several species of anaerobic methanotrophic (ANME) archaea of the *Methanoperedens* genus [3]. A search for other ECEs associated with these archaea also led to the recent discovery of large plasmids found in members of the same genus, yet a distinct clade of *Methanoperedens* species [4].

ANME perform anaerobic oxidation of methane (AOM) by using the reverse methanogenesis pathway [5]. One outstanding feature of Borgs is that they carry genes that encode proteins involved in key steps of their hosts’ metabolism. Remarkably, some Borgs encode the methyl-CoM reductase, all encode multiheme cytochromes (MHCs) that relay electrons onto extracellular terminal electron acceptors, and others encode nitrogenase used for nitrogen fixation [3]. The metabolic potential of different Borgs varies, suggesting diverse modes of interplay between Borgs and their hosts.

Borg genomes share a conserved genomic architecture that is very distinct from *Methanoperedens* chromosomes and plasmids of *Methanoperedens*. They are linear and large, with genome sizes for the first described examples ranging from 0.66 to 0.92 Mbp, thus exceeding known archaeal virus genome sizes by far. This places them in the range of giant and large eukaryotic double-stranded DNA viruses from the nucleocytoplasmic large DNA virus (NCLDV), with genome sizes that can exceed 2.5 Mbp [6]. Linearity also occurs in some virus genomes [7] and in eukaryotic chromosomes (*Saccharomyces*) as well as large linear plasmids of bacteria (*Streptomyces*, *Micrococcus luteus*) [8–10]. The Borg genomes reported to date have a large and a small replichore, and both carry virtually all genes only on one strand. Borg genomes are terminated by long inverted repeats, and nucleotide tandem repeats (TRs) are scattered throughout their entire genomes. These TR sequences follow a head-to-tail pattern, occur in both intergenic and genic regions, and the units are perfectly repeated without single nucleotide polymorphisms (SNPs).

Since these perfect nucleotide TRs are a key common feature of Borgs, we decided to investigate them further to shed light on their potential functions. Here, we analyze TR regions in Borg genomes that comprise ≥50 nucleotides in length and include ≥3 repeat units. We found that DNA sequence assembly algorithms often collapse TR regions and that TRs frequently terminate contigs. This is not surprising, given that repeats in general are a well-known cause for errors in assemblies [7–10]. Thus, we augmented the four existing manually curated Borg genomes by manually curating six new Borg genomes, and twelve additional Borg contigs, to more completely uncover TRs. We found that TRs within open reading frames (ORFs) did not disrupt ORFs, were of lengths divisible by three, and thus form amino acid tandem repeats (perfect aaTRs within proteins). We then bioinformatically analyzed these aaTRs alone and in conjunction with the proteins they arise in. The high frequency, abundance, and within population evolutionary dynamics of repetitive sequences suggest that they are fast evolving and have important biological functions.

## 2. Results

### 2.1. Genome curation and TR analysis

#### 2.1.1. Curation of repetitive DNA sequences to complete Borg genomes

**Fig 1:**
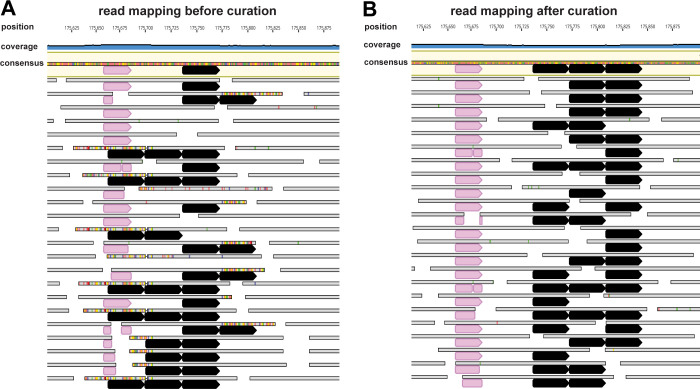
Reads mapped to a de novo assembly before and after curation. Reads are shown as grey bars below the consensus sequence. SNPs in reads are highlighted in color. The pink segments mark a unique sequence that was used to place misplaced reads correctly and the nucleotide repeat unit is shown as black segments. (**A**) A large number of SNPs indicates a local assembly error associated with a collapsed repeat region, resulting in a consensus sequence with one repeat unit. (**B**) The consensus sequence has three repeat units after curation.

We reconstructed and manually curated six new Borg genomes to completion (see Methods; **Table 1**). Curation included correction of local errors where the automatically generated assemblies collapsed regions, or incorporated the wrong number of repeat units. These regions were identified visually based on elevated incidence of SNPs (**Fig 1A**) that clearly indicated that the region was misassembled (**Fig 1B**). All gaps created during the scaffolding step of the assembly were filled and genome fragments extended by making use of unplaced or misplaced paired reads. In some cases, the extending sequences were used to identify missing genome fragments that were then curated into the final assembly. In a few instances, TR regions exceeded the sequencing insert length so the real number of TR units could not be precisely determined. Resolving the de novo assembly errors unmasked TR regions in the genomes and revealed the aaTRs that these introduce into protein sequences.

**Table 1:** TR statistics across 10 completed Borg genomes.

Borg genome	nucleotide statistics						proteins statistics				TR_ORF_ / TR_intergenic_ (%)	TR_intergenic_ div 3 (%)
	genome size (bp)	# TR	TR regions length (bp)	TR / genome (%)	# repeat units	repeat per bp	# proteins	# TR in ORF	# aaTR proteins	aaTR proteins / proteome (%)		
Black Borg	901,883	56	6,128	0.68	289	0.0003204	1,292	23	26	2.01	41	21
Brown Borg	937,932	50	5,913	0.63	259	0.0002761	1,321	23	21	1.59	46	15
Green Borg	1,094,519	76	10,223	0.93	379	0.0003463	1,517	30	26	1.71	39	15
Lilac Borg	661,708	61	6,733	1.02	299	0.0004519	825	28	22	2.67	46	33
Orange Borg	974,068	53	5,675	0.58	246	0.0002525	1,403	20	17	1.21	38	21
Ochre Borg	725,447	23	2,108	0.29	92	0.0001268	1,170	11	10	0.85	48	25
Rose Borg	623,782	20	2,132	0.34	98	0.0001571	937	12	11	1.17	60	38
Red Borg	685,823	23	3,181	0.46	173	0.0002523	1,034	12	9	0.87	52	18
Sky Borg	763,094	32	3,889	0.51	206	0.0002700	1,138	11	11	0.97	34	14
Purple Borg	918,293	66	6,701	0.73	326	0.0003550	1,321	23	23	1.74	35	28
**average**	828,655	46	5,268	0.62	237	0.0002808	1,196	19	17.6	1.48	44	23
**sum**							11,958		176			

aaTR, amino acid TR; ORF, open reading frame; TR, tandem repeat.

**Fig 2:**
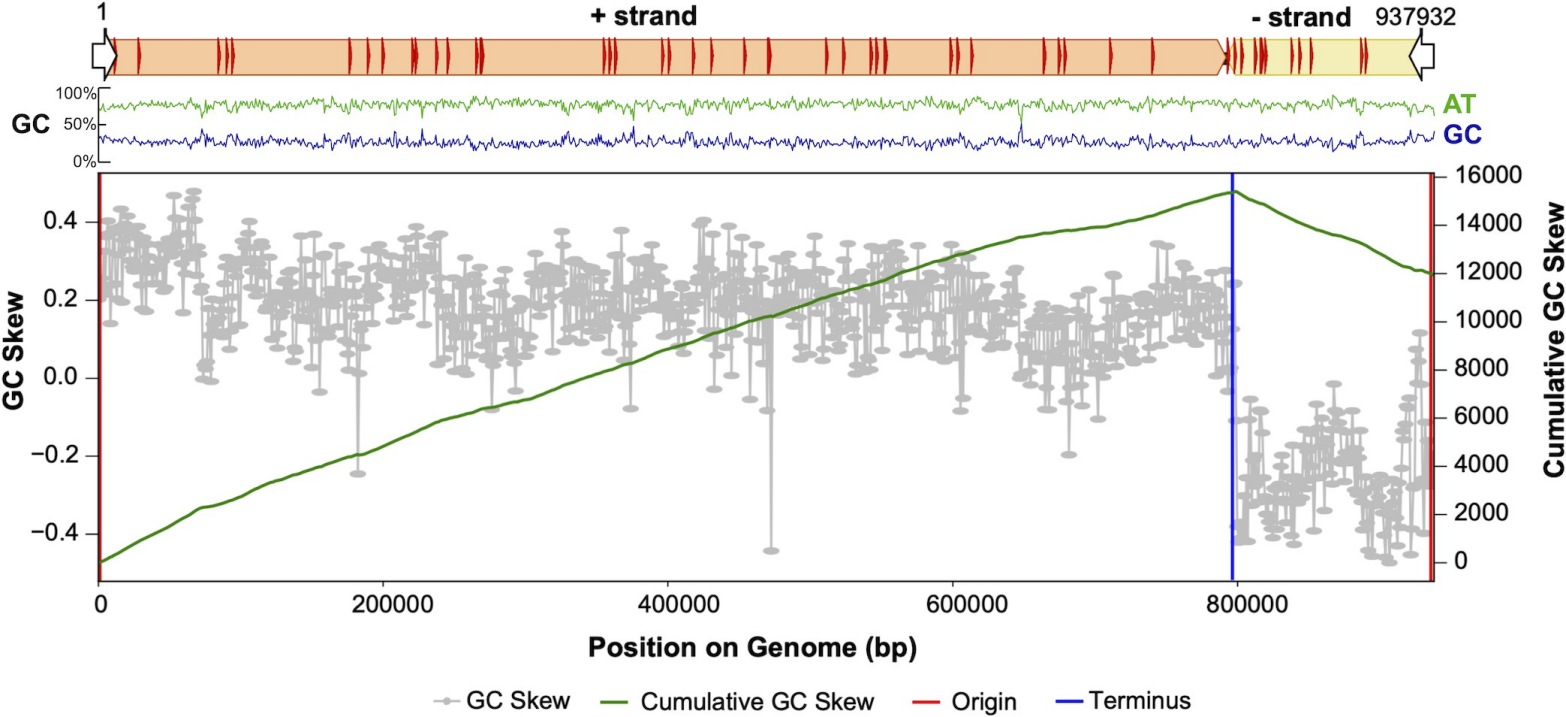
Borg genomes are composed of a large and a small replichore, and their replication is initiated at the termini. Shown is the genome architecture of Brown Borg, including terminal inverted repeats (white arrows), TR regions (red arrows), and GC composition. GC skew and cumulative GC skew show that the genome is replicated from the terminal inverted repeats (origin) to the terminus. The data underlying this Figure can be found in **S1 Table**.

Rose Borg possesses the smallest (623,782 bp), and Green Borg possesses the largest genome (1,094,519 bp). As expected, based on the four previously reported genomes, five of the new genomes are linear, terminated by inverted repeats. Based on GC skew analysis, replication of Borg DNA is initiated at the chromosome ends (**Figs 2 and S1 and S1 Table**). The Red Borg genome contains a repeated sequence that prevented identification of a unique genome assembly path. The variant that generated the expected pattern of GC skew as for the other Borgs was chosen, completing the final set of ten Borg genomes. All genomes but the Green Borg genome are composed of a large and a small replichore; the Green Borg genome has a slightly more complex organization. Each replichore carries essentially all genes only on one strand. Consequently, there are no apparent transcriptional operons. This was mirrored by a low frequency of transcriptional regulators in Borg genomes. Specifically, we only found 0.35 transcriptional regulators per 100 kbp Borg genome, whereas near complete *Methanoperedens* genomes encoded 5.9 in 100 kbp (1 in Rose; 2 in Purple; 3 in Brown, Lilac, Sky, Green; 5 in Purple; 6 in Orange; and none in Ochre and Red Borg versus 53, 53, and 62 in three near-complete *Methanoperedens* genomes SRVP18_hole-7m-from-trench_1_20cm__Methanoperedens-related_44_31,RifSed_csp2_16ft_3_Methanoperedens_45_12,RifSed_csp1_19ft_2_Methanoperedens_44_10; 2.77–2.90 Mbp). We also note that none of the Borgs encode a DNA-dependent RNA polymerase or a TATA-box binding protein.

The previously reported four completed Borg genomes and six new completed Borg genomes were used to evaluate the distribution and features of perfect TRs using a stringent threshold that allowed no mismatch (≥50 nt region and ≥3 TR units). However, during the manual curation, we noticed that some regions had mapped sequencing reads with slight differences in the unit composition, usually a single nucleotide change. This is because reads assigned to a specific Borg likely derive from thousands of genomes, many of which are slightly different to each other.

In the example of a repeat region in a MHC gene, there are two TR unit types that differ by a G → A substitution SNP that is synonymous on the amino acid level. Interestingly, the mixture of TR variants can differ within a Borg population (**S2 Fig**).

To assess whether TR regions were particularly prone to mutation, and, specifically, if there is a bias toward certain SNPs, we analyzed the reads mapped to each Borg genome using inStrain [11]. We detected no SNPs in 3 genomes and extremely few SNPs in 6 genomes (2.2 SNPs across the cumulative TR regions of each genome). The exception was Lilac Borg, which harbored 17 SNPs of which 7 were within the same TR region within an MHC (**S2 Table**). These small numbers preclude any statistical analyses of different mutation incidences in the TR regions compared to the whole genome. Interestingly, we observed an A-bias in the SNPs found in TR regions, and this is primarily at the expense of G (14/18 cases). In contrast, A-SNPs found in non-TR regions were similarly at the cost of G (462/1057 cases) and C (494/1057 cases), but less frequently at the cost of T (101/1057 cases).

#### 2.1.2. Regions with TRs are fast evolving

**Fig 3:**
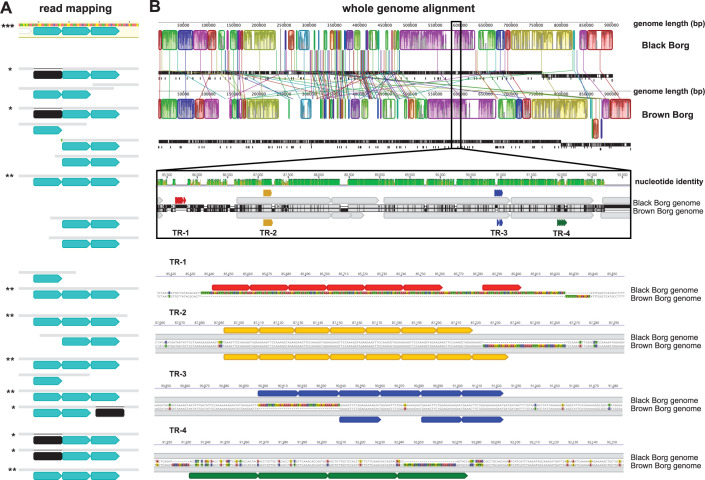
Variability of TR unit numbers within one Borg population and differences in TR regions in two closely related but distinct Borg genomes. **(A)** The consensus sequence of the reads mapped to the curated genome contains three TR units (aqua segments). Reads are shown as grey bars below the consensus sequence (***). Six reads span this region perfectly (**), five reads span this region but are missing one TR unit (*, black segments), nine reads do not span the entire TR region. **(B)** Genome alignment of closely related Black Borg and Brown Borg. Genomic regions that align are depicted as the same-colored collinear blocks in the top panel. A 7-kb region (black box) shows four instances of TRs in these genomes: TR-1 is present in Black Borg but absent in Brown Borg; TR-2 is present in both Borg genomes but with different numbers of TR units; TR-3 is only present in Black Borg, but an imperfect version is present in Brown Borg; TR-4 is only present in Brown Borg. Same color segments show identical TR units; genes are depicted in grey.

The number of TR units in a region sometimes clearly differed within a single Borg population (**Fig 3A**). This implies that the Borg TR regions are fast evolving, much like CRISPR repeat-spacer inventories. Moreover, the repeat loci were rarely conserved in otherwise alignable regions of the most closely related Borgs and were absent from homologous proteins from the host or other homologues in NCBI or our own database ggKbase. This is additional evidence that TR regions formed very recently, after Borgs diverged. A rare case where TR regions are similar occurs in Black and Brown Borgs, which are closely related based on genome alignments (**Fig 3B**). Close inspection of an approximately 7-kbp region in the aligned genomes revealed an 83% sequence identity and four different kinds of TRs. TR-1 is intergenic, is composed of a 20-nt unit repeated six times, followed by a nearly perfect additional unit of 21 nt, then another perfect unit. This intergenic TR is absent in Brown Borg. TR-2 is in an ORF and comprises 18 nt units that are identical in Black and Brown Borgs, where they occur seven and eight times, respectively. TR-3 is also in an ORF and comprises 21 nt units that occur consecutively six times in Black Borg. Brown Borg has two identical units in the same ORF, followed by a sequence that differs by one SNP, then another identical unit. TR-4 is also in an ORF and comprises 36 nt units that occur four times in Brown Borg, but these are absent in the same ORF from Black Borg. Interestingly, the nucleotide sequence from Brown Borg in the vicinity of TR-4 has high nucleotide-level similarity but no TRs.

To quantify the variation in TR unit number and compare it with the count of insertions/deletions in different regions of the Borg genomes, we performed a case study using the genome of Ochre Borg with all reads mapped onto it. We found no instances throughout the entire genome of indels in well-mapped Ochre sequencing reads, except for in the TR regions. For this analysis, we included loci with only two repeat units and found that six of the 27 TR regions showed variation in the repeat unit number. Since many reads start or end within the TR region, they do not provide information regarding the total number of repeat units, hence the true incidence of variation is likely even higher.

#### 2.1.3. TRs are flanked by partial repeats and are enriched in adenine

**Fig 4:**
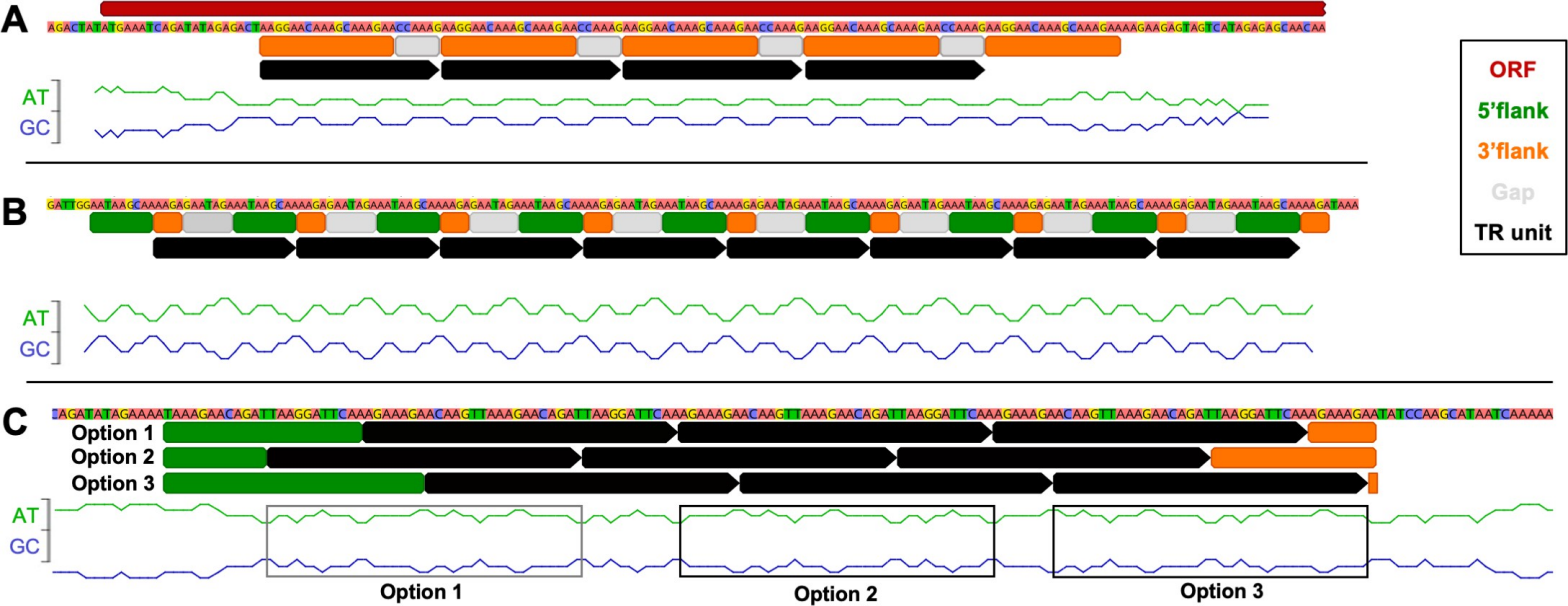
Tandem repeats are often flanked by partial repeat sequences. **(A)** TR regions can occur within ORFs. (**B**) TRs can be in intergenic regions, or the region can start within the end of an ORF. The TR regions are often flanked by partial repeats. (**C**) Different options for the start of a TR region define different flanking partial repeats. The GC graph displays the proportional amount of G or C (or A or T) residues in a sliding window that is set at half of the TR unit length. The data underlying this Figure can be found in **S3 Table**.

To constrain the mechanism behind TR formation in Borg genomes, we assessed characteristics of the TR sequences and their flanking DNA regions. Repetition of sequences with variation in GC versus AT content generally introduces GC/AT-symmetry in regions containing TR units (**Fig 4 and S3 Table**). Offset of the symmetric units and the TRs depends on the exact choice of the repeat unit. The TR regions can be preceded by sequences that are identical to the end of the TR unit and/or followed by sequences that are identical to the start of the TR unit (**Fig 4A and 4B**). These flanking partial repeats are often also adjacent to TR regions that contain only two repeats (and were excluded from statistics). Often the middle of the TR unit is not in either of the 5′ or 3′ partial repeats. When there are different choices for the repeat unit there are slightly different partial repeats that flank the TR sequences (**Fig 4C**).

The nucleotide composition of the ORF-carrying strands on both replichores was similar, with nucleotide frequencies of A>T>G>C (38.4%, 28.8%, 20.3%, 12.5%). The nucleotide composition of all TR units compared to this overall composition showed a strong bias towards A (48.4%), while all other nucleotides were depleted (T 23.7%, G 17.1%, C 10.8%) (**S4 Table**). This A-bias is analogous to the A-bias observed in SNPs within TR regions (**S2 Table**). To establish that the differences in nucleotide composition of the TRs versus all nucleotides are significant, we performed a nonparametric Kruskal–Wallis test [12] and a false discovery rate (FDR) correction according to Benjamini–Yekutieli [13]. This revealed that the compositional bias between the nucleotide TRs and non-TR sequences is of high statistical significance with corrected *p*-values of 9.78 × 10^−68^, 1.95 × 10^−41^, 1.46 × 10^−29^, 1.51 × 10^−23^ for A, T, G, C. This bias was less pronounced in TRs within ORFs (A 46.3%), and these TRs were also enriched in C (15.5%), whereas T and G were depleted (18.6%, 19.6%) (corrected *p*-values of 1.39 × 10^−18^, 5.06 × 10^−58^, 8.40 × 10^−3^, 2.16 × 10^−3^ for A, T, G, C) (**S4 Table**). The fact that this compositional bias is observed in both genes, intergenic regions and in flanking partial repeats suggests that the A enrichment is important for the process that forms the repeats. Predicting the RNA secondary structure of the nucleotide TRs with RNAfold [14] revealed that some are predicted to form loops and others form hairpins.

We searched for polymerases in Borg genomes, given their potential relevance for repeat formation [15]. We found that all Borgs encode at least one DNA polymerase and phylogenetic placement within reference sequences [16] revealed that there are two types of PolBs encoded in the Borgs: the B2 clade and the B9 clade (**S3 Fig**). The DNA PolB9 encompasses a predicted 3′ to 5′ exonuclease and was found in each complete Borg genome. The amino acid sequences are very similar, suggesting a high mode of conservation for these particular proteins.

#### 2.1.4. TRs are rare in *Methanoperedens* and introduce aaTRs in Borg ORFs

To shed light on the role and function of the TRs, we surveyed their occurrence across all ten complete Borg genomes. We found 460 regions that make up 0.62% of the average Borg genome (**Table 1**). Draft *Methanoperedens* metagenomes on the other hand only have 1 to 4 TR regions, making up ≤0.01% of the metagenomes (0.0099% in SRVP18_hole-7m-from-trench_1_20cm__Methanoperedens-related_44_31, 0.0021% RifSed_csp2_16ft_3_Methanoperedens_45_12, 0.0018% in RifSed_csp1_19ft_2_Methanoperedens_44_10), suggesting that TR formation is highly genome specific. Approximately half (43% to 65%) of the Borg TRs were located within ORFs. All TRs within ORFs had unit lengths that are divisible by three, so these repeats do not disrupt reading frames. They result in amino acid tandem repeats that we refer to as aaTRs (and aaTR-proteins). Only 14% to 38% of intergenic TR unit lengths were divisible by three (**S4 Fig**). Sometimes, several different TRs occurred within the same ORF, so the dataset comprised 214 aaTRs in 178 individual proteins from 10 Borg genomes. Fifteen aaTR-proteins were not perfect TRs on the nucleotide level, yet almost all were imperfect due to the occurrence of single SNPs. The TR nucleotide sequences are virtually all unique within each Borg genome and are very rarely shared between Borg genomes, with only five cases of identical TR units in the same genome (Green Borg TR 16 and 19 and TR 17 and 21; **S14 Table**) or shared between Borgs (Black Borg TR 35 and Brown Borg TR 31, Black Borg TR 43 and Brown Borg TR 38, Purple Borg TR 21 and Ochre Borg 13). Upon inspection of aaTR-proteins, we found a different codon usage in genes with TRs relative to all Borg genes. As expected, based on the relatively A-rich composition of repeat regions, the aaTR-bearing ORFs generally favored incorporation of codons containing A. Codons containing T in any position were generally depleted in aaTR regions. Codons with C in position 1 or 2 of the triplett, and G in position 1 were enriched in aaTRs, but codons with C or G in other positions or combinations of positions 1 to 3 were depleted (**S5A Fig and S5 Table**). The most frequent codons in aaTRs were CCA (encoding proline) and ACA (encoding threonine) with an up to 3-fold higher frequency in aaTRs than in non-aaTR regions. The most statistically enriched codons in TRs were GAT and AAA (encoding aspartate and lysine with corrected *p*-values of 1.63 × 10^−57^ and 1.66 × 10^−52^), and most statistically depleted codons in TRs were ATG and TTT (start codon and phenylalanine with corrected *p*-values of 6.43 × 10^−104^ and 6.67 × 10^−100^) (**S5 Table**). Seven codons were completely absent in aaTRs, namely, TGA, TAA, and TAG (stop codons), CGA, CGT, and CGG (encoding arginine), TTC (phenylalanine), and TGC (cysteine) (**S5B Fig**).

### 2.2. Biophysical properties of aaTRs

#### 2.2.1. Amino acid frequency in aaTRs

**Fig 5:**
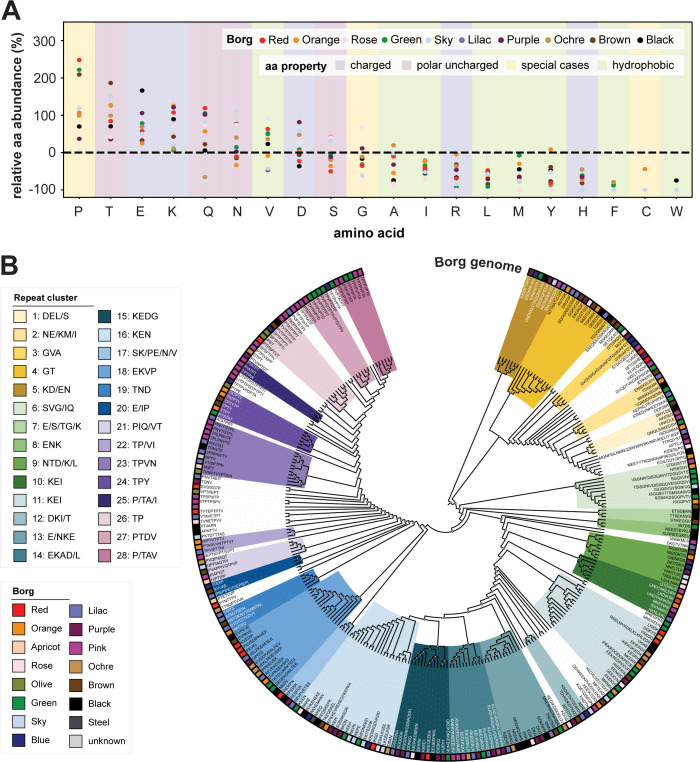
Relative amino acid abundance in aaTRs and hierarchical clustering of triple aaTR units. **(A)** Relative aa abundance in aaTRs compared to all proteins. (**B)** Repeat units were hierarchically clustered based on triple aaTR units. Clusters with ≥3 aaTR members were named by amino acids that form the aaTR unit in descending order (threshold ≥10%). The color strip shows in which Borg genome each aaTR is encoded. The data underlying this Figure can be found in **S7** and **S8 Tables**.

We closely examined the predicted biophysical and biochemical properties of the single repeat units from all ten Borg genomes and 37 additional proteins from curated Borg contigs. This final dataset comprised 215 Borg aaTR-proteins and 306 repeat regions (**S6 and S7 Tables**). Proline, threonine, glutamate, and lysine were particularly enriched across all Borgs, while tryptophan, cysteine, and phenylalanine were almost absent (**Fig 5A and S8 Table**). We noticed that disorder-promoting amino acids were enriched in the aaTRs and order-promoting amino acids were depleted (**S6 Fig**).

Hierarchical clustering of triple aaTR units revealed 28 aaTR clusters, each cluster composed of aaTRs with very similar amino acid composition and frequency (**Fig 5B and S7 Table**). Many aaTR units possessed nearly equal numbers of the charged amino acids K and E (polyampholyte repeat cluster); others were enriched in P/T/S, which are potential sites of posttranslational modification. From this, we conclude that there are distinct and related groups of aaTRs.

#### 2.2.2. aaTRs are enriched in extracellular proteins, and most aaTRs are intrinsically disordered

Strikingly, 32.1% (69/215) of aaTR-proteins have extracellular regions and 30.2% have transmembrane helices (TMHs), whereas only 18.6% (2,230/11,995) and 16.7% of all Borg proteins have these features. Consistently, aaTR-proteins are highly enriched in signal peptides (16.7% of aaTR-proteins compared to 4.0% of all Borg proteins).

**Fig 6:**
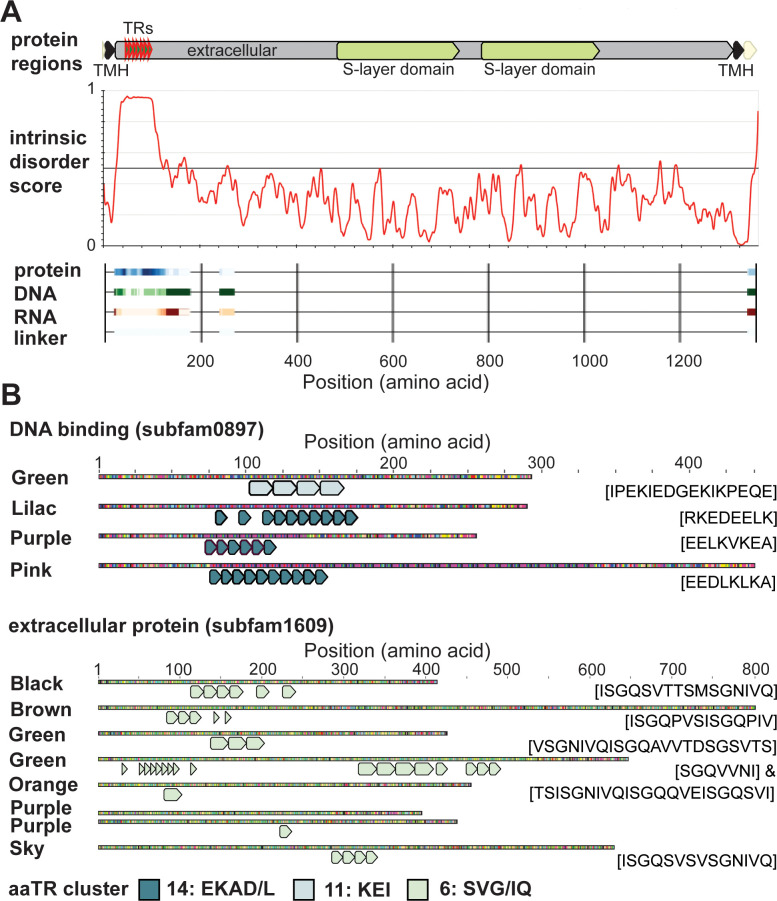
aaTRs introduce intrinsic disorder, are predicted binding sites, and comprise similar amino acids in homologous Borg proteins. **(A)** Domain architecture of S-layer protein from Green Borg. The aaTR region is intrinsically disordered (predicted with IUPred3; [28]), and the IDR regions are predicted binding sites for proteins, DNA, RNA, or a linker region (predicted with flDPnn [60]; probability of feature increases with color intensity). (**B)** All or most members of two protein subfamilies have aaTRs. The aaTRs within a protein subfamily are from one or two related aaTR clusters. The amino acid sequence of each aaTR unit is shown in brackets.

To further investigate the observation that aaTRs particularly often contain disorder-promoting amino acids, we assessed the prominence of intrinsically disordered regions (IDRs) in aaTR-proteins versus all Borg proteins. IDRs are polypeptide segments that are characterized by a lack of a well-defined 3D structure [17]. Remarkably, 62.8% aaTR-proteins contained at least one IDR (≥15 amino acids), whereas only 5.6% of all Borg proteins contained an IDR. The relative length of the IDR regions varied from 2.6% to 100% and 1.2% to 100% in both aaTR-proteins and non-aaTR-proteins (**S7 Fig and S9 Table**). In aaTR-proteins, the IDRs almost always corresponded to the TR region, and *Methanoperedens* homologues did not have IDRs (**Fig 6A**). Thus, we hypothesize that the TRs in ORFs mostly lead to the creation of new IDRs in existing proteins.

Forty proteins possessed multiple repeat regions ranging from two to five aaTR regions (**S10 Table**), and the aaTRs were often located at the N- or C-termini or between domains. Some of these regions fall into the same or a similar aaTR cluster, whereas others are distinct and often located in a completely different region of the polypeptide chain. In 22 cases, the aaTRs make up the majority (50% to 96%) of the predicted proteins, and many of these are small proteins (14 proteins are <100 aa) (**S11 Table**). The repeat units of these proteins are diverse, but clusters enriched in K, E, and I (cluster 10 and 11) were particularly frequent (6/18). Although it is possible that these are wrongly predicted genes in intergenic regions, they may also be de novo repeat peptides. Their existence as real proteins is supported by the observation that they possess a start codon (or alternative start codon), the TR units are divisible by three and thus introduce aaTRs, and by the fact that four have signal peptides and four have functional annotations (linked to apolipoprotein, proline-rich domains, and a glycoprotein domain).

### 2.3. Functions of aaTR-proteins

#### 2.3.1. Similar aaTRs fulfill similar predicted functions, and functionally related proteins have similar aaTRs

To investigate which functions aaTR-proteins have, and if functionally related proteins possess similar aaTRs, we performed protein family clustering of 11,995 Borg proteins. In brief, we clustered proteins using the fast and sensitive protein sequence searching software MMseqs2 [18] in an all-versus-all approach to define protein subclusters based on amino acid similarities. Based on the alignments of the subfamily members, HMMs were generated for each subfamily using HHblits [19]. These HMMs were then functionally annotated by an HMM-HMM comparison with the PFAM database using HHSearch [20]. This resulted in 85% of Borg proteins being grouped into 1,890 subfamilies and 80% (172/215) of the aaTR-proteins clustered into 112 subfamilies (**S6 Table**). Based on the annotations for the individual protein members of each subfamily and the annotation of the HMM of the subfamily, protein subfamilies were manually grouped in terms of function. The functional landscape of the subfamilies ranged from carbon metabolism (18 proteins), cell and protein architecture and scaffolding (9), nucleotide processing (15), transcription-associated proteins (8), redox (8), signaling (11), transport (3), stress response (2), to protein processing (1) (**Table 2**).

**Table 2:** Functional annotations for protein subfamilies with aaTR-bearing protein members. Proteins were manually placed into functional categories based on the pfam annotation of the subfamily. Single aaTR units are listed as well as in which aaTR cluster they fall. Some aaTRs did not fall into clusters (n.c., no cluster), and some proteins have two or more aaTRs, which fall into one or two aaTR clusters.

Borg	subfamily	pfam	pfam annotation	functional category	subcategory	repeat unit	aaTR cluster	aaTR cluster 2
**Red**	subfam0056	PF01670	Glyco_hydro_12	**carbon metabolism**	carbohydrate	PTPSPTVT	26: TP	
**Lilac**	subfam0140	PF00704	Glyco_hydro_18			NTTNATVLGQ	4: GT	
**Pink**	subfam0155	PF05219|PF08323|PF12000|PF00343	DREV Glyco_transf_5 Glyco_trans_4_3 Phosphorylase			HNKDLE	1: DEL/S	
**Lilac**	subfam0184	PF05693	Glycogen_syn			NSEHE	13: E/NKE	
**Black**	subfam0196	PF06560	GPI			KPISNK	17: SK/PE/N/V	
**Pink**	subfam0259	PF00295	Glyco_hydro_28			ENLDKKQDN	5: KD/EN	
**Brown**	subfam0639	PF10633	NPCBM_assoc			STIKEGGV	7: E/S/TG/K	
**Green**	subfam0966	PF11735	CAP59_mtransfer			KEDAVKEGTEKP	n.c.	
**Pink**	subfam1073	PF16363	GDP_Man_Dehyd			VNNKMLS	n.c.	
**Brown**	subfam1315	PF01233	NMT			SKDTGV	4: GT	
**Green**	subfam1611	PF14519	Macro_2			MNESNKDNIGDK	13: E/NKE	
**Olive**	subfam1644	PF15421	Polysacc_deac_3			PTPE, VPTPEPT	n.c.	
**Rose**	subfam1766	PF03142|PF13506	Chitin_synth_2 Glyco_transf_21			KDGS	n.c.	
**Orange**	subfam1074	PF00278	Orn_DAP_Arg_deC		amino acid	DGKYS	11: KEI	
**Red**	subfam0779	PF00708	Acylphosphatase		carbon and energy	KLVEGQN	n.c.	
**Green**	subfam1586	PF00285	Citrate_synt			MNEGFKN	n.c.	
**Green**	subfam0998	PF09712	PHA_synth_III_E			DESEKGINLTEK	18: EKVP	
**Purple**						SESPVK	17: SK/PE/N/V	
**Brown**	subfam0274	PF06005	ZapB	**architecture**	cell division	KVVAE	20: E/IP	
**Black**	subfam0452	PF08308	PEGA			RSELMYKDKLKLKRLEQE	11: KEI	
**Black**	subfam0483	PF01145	Band_7		anchor + proteolysis	EEKKE	15: KEDG	
**Sky**	subfam0493	PF08308|PF08308	PEGA PEGA			YNNTLSD	9: NTD/K/L	
**Pink**						IITPTPSPV, TPSPIIITP	n.c.	
**Green**	subfam0771	PF07752|PF07752	S-layer S-layer			TPTPVPTD	27: PTDV	
**Pink**	subfam1419	PF13809	Tubulin_2		polymer formation	KQEVQSEQN	n.c.	
**Purple**						QIENKDS	9: NTD/K/L	
**Lilac**						AEVEKGVEK	18: EKVP	
**Lilac**	subfam0601	PF14582	Metallophos_3	**nucleotide processing**		GNN	13: E/NKE	
**Ochre**	subfam0875	PF18741	MTES_1575		transcription	EKIEKGII	11: KEI	
**Green**						EKEKVI	11: KEI	
**Lilac**	subfam0897	PF09397	FtsK_gamma		chromosome segregation	RKEDEELK	14: EKAD/L	
**Pink**						KVKAEEELKVKAE, KAEKVKAEEELKV, EEDLKLKA	14: EKAD/L	
**Purple**						EELKVKEA	14: EKAD/L	
**Green**						IPEKIEDGEKIKPEQE, KKEEI	11: KEI	15: KEDG
**Purple**	subfam1236	PF18516	RuvC_1		DNA processing	NKDN	13: E/NKE	
**Steel**	subfam1261	PF17209	Hfq			NKEQ	16: KEN	
**Black**						KEQSKEPK	16: KEN	
**Orange**						EPKKEVREKEQVKEPKK	16: KEN	
**Brown**						TKEQSKEL	16: KEN	
**Ochre**						KEQIKDSKKEQV	n.c.	
**Lilac**	subfam1747	PF04139	Rad9		DNA repair	DRGGF, TGIGEI	4: GT	
**Ochre**	subfam1533	PF05491|PF11023	RuvB_C DUF2614			EHEKG	13: E/NKE	
**Purple**	subfam1678	PF00382	TFIIB	**transcription**		IEKNMRD	10: KEI	
**Purple**	subfam1779					NNELTLK	9: NTD/K/L	
**Lilac**	subfam0108	PF11023	DUF2614		?	AEPIKLPEVPIIPKE, VELPKEAESLK	18: EKVP	
**Black**						EVPKVE, EVPKVE	18: EKVP	
**Brown**						EVPKVE, EVPKVE	18: EKVP	
**Green**						TKTELVAPKTEDI	18: EKVP	
**Orange**						DVPKSEHSKS	18: EKVP	
**Red**						KVEDIP, PKVEL, PKVEDIPKVEP	18: EKVP	
**Olive**	subfam0267	PF02335|PF03264|PF02335|PF03264|PF03264|PF13447	Cytochrom_C552 Cytochrom_NNT Cytochrom_C552 Cytochrom_NNT Cytochrom_NNT Multi-haem_cyto	**redox**		SPTPTPTI, SPTPTPTI	26: TP	
**Blue**						SPTPTPTI, SPTPTPTI	26: TP	
**Brown**	subfam0540	PF00127	Copper-bind			NSTSKNVTKINNTKL	19: TND	
**Orange**						INNTED	19: TND	
**Black**	subfam0608	PF02335|PF02085	Cytochrom_C552 Cytochrom_CIII			TTSEKNVS	7: E/S/TG/K	
**Rose**	subfam1117	PF00248	Aldo_ket_red			LSVLNNN	9: NTD/K/L	
**Orange**	subfam1659	PF00127|PF06525	Copper-bind SoxE			ISDIGNMA	n.c.	
**Pink**	subfam1791	PF13447|PF13750	Multi-haem_cyto Big_3_3			TPTPIPTD	27: PTDV	
**Sky**	subfam0496	PF00005|PF06068	ABC_tran TIP49	**transport**		NEPE, NEPK	16: KEN	
**Orange**	subfam0795	PF17795|PF11978	Vault_3 MVP_shoulder		ribonucleoprotein	NKDDKTG	9: NTD/K/L	
**Ochre**	subfam1027	PF05753	TRAP_beta			MKNKII	11: KEI	
**Lilac**	subfam1529	PF16968	TadZ_N	**signalling**		DTDITDTKDVT	19: TND	
**Green**	subfam1750	PF04185	Phosphoesterase			PVPTATPT, TPVPTDTP, PVPTDTPTPVPTDTPV	27: PTDV	
**Green**						TPTPIPTA	27: PTDV	
**Pink**	subfam1773	PF09788	Tmemb_55A		phosphatase	2x KLADVRADKLADERAN, PIAPIQT	14: EKAD/L	21: PIQ/VT
**Purple**						KLADERAD, 2x KLADVRADKLADERAN	14: EKAD/L	
**Brown**						PVAPPIVQTPVP	21: PIQ/VT	
**Ochre**						EEQDRKLR, PVQPVAQT	14: EKAD/L	21: PIQ/VT
**Sky**						LKQDLESR, QPPIAQTPI	16: KEN	21: PIQ/VT
**Green**						AEEQAKKF	14: EKAD/L	
**Lilac**						2x KLADERAD	14: EKAD/L	
**Rose**						ESKLKQDL	16: KEN	
**Rose**	subfam0581	PF05957	DUF883	**stress response**		EGK	13: E/NKE	
**Black**	subfam1546	PF04055|PF12544	Radical_SAM LAM_C		compatible solutes	ENK	13: E/NKE	
**Black**	subfam1168	PF14890|PF05203	Intein_splicing Hom_end_hint	**protein processing**		NEENE	13: E/NKE	
**Rose**	subfam0079	PF04009	DUF356	**?**		GTDIIKQV	3: GVA	
**Red**	subfam0307	PF18546	MetOD1			TGAVR	n.c.	
**Lilac**	subfam1290	PF14205	Cys_rich_KTR			KKNLKRL	10: KEI	

Screening the aaTR-proteins with the eukaryotic linear motif (ELM) resource for functional sites in proteins (http://elm.eu.org/) unmasked that the aaTRs within them have similarity to a plethora of different short linear motifs (SLiMs). SLiMs are composed of three to ten consecutive amino acids [21], which are used by eukaryotic cells as cleavage sites, degradation sites, docking sites, ligand binding sites, posttranslational modification sites, and targeting sites [22]. We found that aaTRs from the same aaTR cluster or related aaTR clusters often had matching functional predictions (see examples in **S7 Table**).

Fifteen protein subfamilies were enriched in aaTR-proteins, which included subfamilies functionally annotated as transmembrane phosphatases, ribonucleoproteins, phosphoesterases, zinc-ribbon proteins, and DNA-binding proteins; the other subfamilies had no functional annotations (**S6 Table**). Members of the same protein subfamily often had similar aaTRs. For example, a subfamily of DNA binding proteins (subfam0897) only comprises aaTR-bearing members, all of which have aaTRs that are unique in nucleotide sequence (**S8 Fig**), yet of the same or related repeat clusters (**Fig 6B**). These aaTRs form a predicted coil structure or an IDR and resemble SLiMs that play a role in ligand binding, degradation, and targeting (**S7 Table**). Similarly, subfam1609 comprises eight members, five of which are aaTR-proteins, and, despite all being only distant homologues (highest aa identity is 67%), the aaTRs are encoded by unique nucleotide TR units (**S8 Fig**) but belong to the same aaTR cluster (SVG/IQ), which corresponds to predicted modification (phosphorylation) and ligand-binding sites (**Fig 6B** and **S7 Table**).

#### 2.3.2. aaTR-proteins responsible for cell integrity/stability and surface decoration

Several functionally related but phylogenetically unrelated proteins that are responsible for cell wall architecture (PEGA and S-layer) and decoration (glycosyltransferases and glycosyl hydrolases) have aaTRs. The aaTRs of the PEGA proteins resemble predicted ligand binding, docking, and modification (phosphorylation) sites, and the aaTRs of the S-layer resemble proteolysis-initiating degrons [23]. The aaTRs of glycosyl hydrolases resemble modification and ligand binding sites, and the aaTRs of glycosyl transferases resemble degrons (**S7 Table**). Some Borgs also possess an aaTR in tubulins, which are proteins required for cell division [24]. These aaTRs resemble SLiMs that are potential docking and cleavage sites and/or initiate proteasomal degradation. Importantly, these SliM-bearing aaTRs are absent in non-Borg homologues.

### 2.4. Case studies of aaTR-proteins: Ribonucleoproteins, MHCs, and a conserved TR hotspot

To further investigate proteins with the same function that evolve similar but not identical aaTRs, we performed in-depth analyses of two distinct types of proteins with a known function and a conserved region across Borgs comprising multiple aaTR-proteins.

#### 2.4.1. Ribonucleoproteins

**Fig 7:**
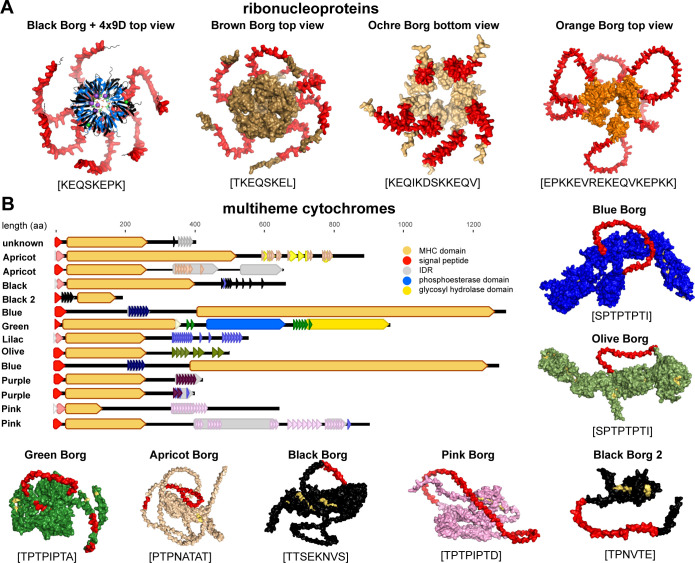
Predicted structures of aaTR-bearing ribonucleoproteins and multiheme cytochromes. (**A**) Sm ribonucleoprotein from Black Borg was superimposed on Sm from *M*. *jannaschii* (blue, PDB: 4X9D with UMP RNA + ions, magenta). (**B**) MHC domain architecture and predicted structures. aaTR regions in predicted structures are highlighted in red, and amino acid sequence of each aaTR unit is shown in brackets below each structure. Structures were predicted with AlphaFold2.

Most Borgs encode Sm ribonucleoproteins, which are archaeal homologues of bacterial Hfq and eukaryotic Sm/Sm-like (Lsm) proteins. These proteins are implicated in versatile functions such as RNA-processing and stability, and the protein monomers form a stable hexameric or heptameric ring-shaped particle [25,26]. We found 19 Borg Sm ribonucleoproteins (**S12 Table**), five possess aaTRs, and one additional sequence from Grey Borg has a near-perfect aaTR. The aaTRs are always located at the N-terminus, preserving the conserved Sm1 and Sm2 RNA-binding motifs (**S9A Fig**). The aaTR units contain 4, 8, 12, or 17 amino acids, have the same aa character, and are predicted SLiMs that facilitate docking or resemble degrons (**S9B Fig and S12 Table**). An initial homology-based structural search of the aaTR-Sm from Black Borg identified the bacterial Hfq of *Pseudomonas aeruginosa* (PDB: 4MML, aa identity: 26%) that forms a homohexameric ring structure. Due to no alignable template in the database for the aaTR, the model failed to predict a structure of this N-terminal region. Remodeling with AlphaFold2 predicted the structure of the Sm core that formed a hexameric ring with long loops extending at the distal side of the protein (**Fig 7**). This unstructured region corresponds to the aaTR region and matches the prediction of MobiDBLite [27] and IUPred3 [28] that the aaTR is an IDR. Modeling of the other four aaTR-Sm proteins also showed N-terminal unstructured extensions corresponding to the aaTRs, which were similarly predicted IDRs (**S9C Fig**).

#### 2.4.2. Extracellular electron transferring MHCs

All Borgs encode MHCs, which are particularly important and abundant in *Methanoperedens* as they mediate the final electron transfer from methane metabolism to an external electron acceptor [29]. We found 14 MHCs with aaTRs in Borg genomes. All of these are predicted to be located extracellularly, some possess a membrane anchor, and they range from 20.5 kDa (190 aa) to 137.5 kDa (1,293 aa) and contain up to 30 heme binding sites (**Fig 7**). The proteins are thus clearly distinct in sequence and domain architecture. The aaTRs are located at different sites in the polypeptides but never disrupt existing functional domains. Yet remarkably, the aaTRs belong to identical or similar repeat clusters that are consistently enriched in T and P and resemble ligand binding, docking, and modification sites (**S13 Table**). The aaTRs are mostly IDRs, which are predicted to form unstructured extensions that protrude from the folded protein core.

#### 2.4.3. A conserved aaTR hotspot

**Fig 8:**
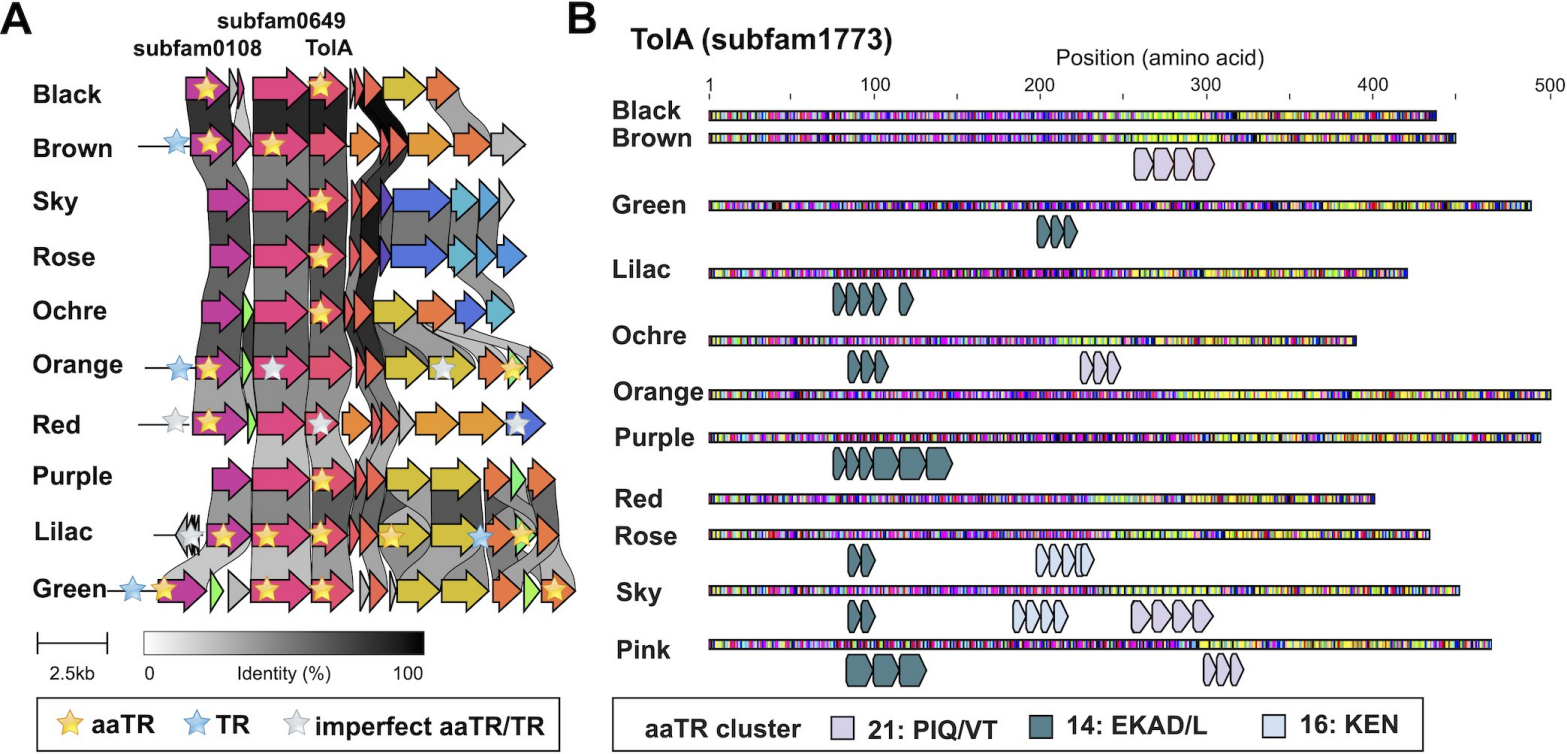
A conserved genetic region is a hotspot for tandem repeats. **(A**) Gene cluster alignment of a conserved region in all Borg genomes that encompasses TolA. The alignment is based on amino acid identity and was generated with clinker [48]. (**B**) Most TolA homologues have at least one aaTR region. The aaTR units are depicted as arrays below each sequence, and regions with two aaTR units only are shown as well.

There is a region in all complete Borg genomes that is a hotspot for TRs with up to five aaTR-bearing proteins (**Fig 8A**). It includes a gene encoding subfam1773, which we refer to as cell envelope integrity protein TolA. Best blastp and structural hits are TolA from, e.g., the pathogenic bacterium *Leptospira sarikeiensis* (48% aa identity with WP_167882360) and YgfJ from the pathogen *Salmonella typhimurium* (PDB: 2JRP). Eight TolA proteins have unique aaTRs, and some carry additional aaTR units shared by other Borgs (e.g., Ochre Borg repeat units are found in Rose Borg and Purple Borg) (**Fig 8B**). These aaTRs are similar in amino acid composition and they are predominantly predicted degradation, docking, and ligand binding sites (**S6 and S7 Tables**). The regions encode two other conserved protein subfamilies with aaTR-bearing members: subfam0649 lacking functional annotation and a subfamily of zinc-ribbon proteins (subfam0108), which usually form interaction modules with nucleic acids, proteins, or metabolites. The subfam0108 proteins possess seven distinct aaTR units, six of which fall into the same repeat cluster (**S6 and S7 Tables**). Additional aaTR-bearing proteins in the same context are a glycogen synthase/glycosyl transferase (subfam0184) in Black Borg and Green Borg and proteins of unknown function (subfam1382) in Lilac Borg and Orange Borg, and a hypothetical protein in Green Borg.

## 3. Discussion

Tandem nucleotide repeats in Borg genomes likely are formed and evolve independently, as evidenced by the essentially unique sequences of TR units in each region within and among Borg genomes. They evolve rapidly, based on differences in the alignable regions of the most closely related Borgs (e.g., Black and Brown Borgs) and variability in repeat numbers within a single Borg population. This parallels the rapid evolution of eukaryotic variable number TRs (VNTRs). VNTRs are widespread and linked to neuropathological disorders (caused by the accumulation of short TRs) [30], gene silencing [31], and rapid morphological variation [32]. In bacterial genomes, highly repeated sequences are less common, and TR variations are linked to immune evasion, cell-pathogen specificity (definition of which cells/tissues/host pathogens/viruses can infect), and stress tolerance [33,34]. They are relatively common in a few archaeal genomes, but the functions there remain uncertain.

Previously proposed mechanisms for the expansion and retraction of VNTRs implicate various DNA transactions, including replication, transcription, repair, and recombination [15]. Replication slippage is one explanation for the origin and evolution of repeat loci [33]. This mechanism involves a pausing of the DNA polymerase and dissociation from the TR region, followed by DNA reannealing. Realignment of the newly synthesized strand can be out-of-register on the template strand, leading to propagation/retraction of the TRs. At this time, it is unclear whether the TRs are introduced by Borg or *Methanoperedens* machinery. If the former, it is notable that we found that all ten Borgs encode a highly conserved DNA PolB9, a clade of uncharacterized polymerases that have only been reported from metagenomic datasets [16]. Archaeal DNA polymerases B and D have been shown to slip during replication of TR ssDNA regions [35]. Thus, the Borg PolB9 could be responsible for TR propagation, possibly triggered by noncanonical secondary structures formed by the TR DNA or its upstream region [35].

Given the shared nucleotide characteristics of genic and intergenic TR regions (perfection, A bias), we infer that they form by the same mechanism. Even if TRs are introduced perfectly, one would expect some of them to have accumulated mutations, unless perfection is strongly selected for and ensured through a repair mechanism, or unless they all formed very recently. The latter is consistent with other evidence that TR regions are fast evolving, namely the variability in TR unit number within a Borg population.

In many cases, the sequences that flank the TR regions are partial repeats. We suspect that these are seed sequences that were used to initiate TR formation. If these regions were the seeds that gave rise to the TRs, the fact that they are often only parts of the repeat raises a mystery regarding the origin of central regions of these TRs. It is possible that the sequences that served as seeds were subjected to elevated mutation rates, which is supported by the observation of high SNP levels flanking TR regions in the human genome [25,27].

CRISPR repeats suggest a possible alternative to the replication slippage explanation for the origin of TR regions. Like TR regions, CRISPR loci are fast evolving, in that case motivated by the need to counter rapid evolution of bacteriophages to outwit spacer-based immunity [36]. CRISPR repeats are introduced by an integrase that excises the previously added repeat, ligates it to a new spacer, and adds that unit to the expanding locus, filling gaps to recover a double stranded sequence [37]. If a Borg system is involved, the genomes encode many and various nucleases and recombinases, some of which may be responsible for repeat addition. Unfortunately, a complete *Methanoperedens* genome is not available yet, so we cannot confidently assess their capacities for repeat introduction.

As noted above, our analyses revealed a compositional bias in nucleotide TRs towards A on the coding strand of each replichore, primarily at the expense of T. If this reflects mutation, the transversion (conversion of purine to pyrimidine) must happen at the biogenesis of the TR template, as the TRs are faithfully copy-pasted error-free. We tentatively support the alternative explanation that the A-rich nature of the seeds initiated TR formation. A small region that is A-rich, possibly functioning in a manner somewhat analogous to a protospacer adjacent motif (PAM) that binds the CRISPR system nuclease, may localize the machinery involved in repeat formation. We speculate that TR formation could be regulated by distinct methylation patterns of Borg DNA, possibly related to the A-rich character of the seed.

An analysis of the SNPs in TR regions provides another clue regarding A-enrichment in the TRs. Although the small data size precludes statistical analysis, we note that the reads with SNPs are more A-rich than the consensus repeat sequences, and this enrichment is apparently at the expense of G. This could suggest that during Borg genome replication or TR propagation, guanines in TRs are selectively mutated to adenines. This may be reminiscent of G to A transitions observed in human diseases at sites of 5-methylcytosine [38], but much larger datasets will be required to investigate this further.

A key question relates to how stable coexistence of Borgs, with their huge genomes, likely in multicopy [3], and the host *Methanoperedens* cells is achieved. Although it is possible that Borgs direct the functioning and evolution of the hosts upon which they fundamentally depend on for their existence, it is perhaps more likely that *Methanoperedens* controls Borg activity. The mechanisms for regulation of Borg gene expression are unclear, given the absence of obvious operons and the conspicuous lack of transcriptional regulators. As we identified no RNA polymerase and TATA-box binding proteins in any of the ten complete Borg genomes, it is possible that *Methanoperedens* can tightly regulate Borg gene transcription, thus controlling the production and localization of Borg proteins. TRs may be involved in this process. Approximately half of the TR regions were intergenic or have the first unit within or partially within gene ends. These could be (long) noncoding RNAs ((l)ncRNAs), which are known to modulate chromosome structure and function, transcription of neighboring and distant genes, affect RNA stability and translation and serve as architectural RNAs (arcRNAs) [39]. arcRNAs are of particular interest as they are involved in forming RNA-protein condensates, and some arcRNAs possess repeat sequences to accumulate multiple copies of specific proteins and/or multiple copies of RNAs [40].

Virtually all TRs in ORF were of lengths divisible by three, thus do not disrupt ORFs, and their A bias was not as strong as intergenic TRs. This indicates a strong selective pressure to always introduce amino acid repeats and a selection for (or deselection against) specific codons. Bulky or highly reactive amino acids such as cysteine, which could severely interfere with redox homeostasis, were absent. Yet, disorder-promoting amino acids were enriched in aaTRs, and, concomitantly, most aaTR regions were predicted to be IDRs. Thus, we infer that aaTRs could impact protein functioning. While protein homologues from *Methanoperedens* (or other microbial genomes) almost never showed aaTRs, Borg homologues (or functionally related Borg proteins) had aaTRs that were highly similar in aa character, but not identical in DNA sequence. For very closely related Borgs, this may arise because similar DNA regions in Borg genes were seeds for TR formation. However, the presence of aaTRs in different regions of proteins with the same function (e.g., MHCs) indicates a strong selective pressure for specific aaTRs that is constrained by protein function. Overall, we consider the combination of features to be strong evidence that the aaTRs are not random genomic aberrations and those in ORFs can fulfill an important role in protein functioning.

To shed light on the predicted function that the proteins gain by having evolved aaTRs, we screened many of aaTRs for predicted functional sites. Most aaTRs are IDRs, and these are commonly found in SLiMs and vice versa [17]. In the case of the N-terminal aaTRs in the Borg ribonucleoproteins, the long loops that extend from the RNA-binding protein core could serve as docking sites for interaction partners. This is reminiscent of eukaryotic homologues that possess fused C-terminal IDR domains and are part of the spliceosome, a ribonucleoprotein machine that excises introns from eukaryotic pre-mRNAs [41]. The aaTR extension of the Borg ribonucleoproteins may undergo a disorder-to-order transition induced by binding to a yet to be identified protein partner, which could redefine its function in, e.g., pre-mRNA or pre-tRNA processing [42]. The aaTRs associated with MHCs were also predicted to form long, largely unstructured extensions. Their consistent compositional dominance by proline and threonine clearly suggests a shared function and convergent evolution of these aaTRs. Based on the predicted SLiMs within the aaTRs, they could be intraprotein intersections that are glycosylated or phosphorylated, triggering a signaling cascade and conformational changes that lead to a modified electron flow, redox capacity, and, possibly, nature of electron acceptor. Since intrinsic disorder is a common feature of (eukaryotic) hub proteins [43], the aaTRs could also be involved in navigating interprotein interfaces by being docking sites for other MHCs or extracellular oxidoreductases, such as a manganese oxidase that we found in Lilac Borg [3]. We thus propose that the Borg MHCs expand the redox and metabolic capacity of a large MHC network at the cell surface of the host [44] and aaTRs within them may enable a tunable connection to the electron conduction system that is integral to *Methanoperedens’* metabolism.

We found that aaTRs were statistically enriched in Borg proteins with predicted extracellular and membrane localization, another indication that their formation is not a random event. These proteins are typically implicated in cell–cell interactions, transport, protection, and virulence. It is unclear whether Borgs have an existence outside of *Methanoperedens* cells, but the inferred high copy number of some Borgs compared to host cells [3] may point to this possibility. Borg-encoded S-layer and PEGA domain proteins (with and without aaTRs) could potentially encapsulate Borgs and mimic host proteins to evade host defense. Alternatively (or at a different time in their existence), these Borg proteins could be displayed on the *Methanoperedens* cell surface to alter host processes.

If Borgs can exist outside of host cells, they would need the ability to infect *Methanoperedens* cells to replicate (analogous to viruses). Proteins involved in infection and defense are expected to be fast evolving, and TRs may serve as a mechanism to enable this. A hotspot for TR evolution that is conserved across Borgs encodes TolA (and additional conserved hypothetical proteins). TolA is important for cell envelope integrity and is crucial for entry of filamentous bacteriophages that infect *Escherichia coli* or *Vibrio cholerae* [45,46]. TolA is exceptional, as unlike other Borg aaTR-proteins, homologous proteins from *Streptomyces* and *Bacillus* are also repeat proteins with similar aa signatures (EAKQ). The observation that the TolA-containing regions in Borgs are conserved, fast evolving, and under strong selective pressure could be consistent with a role in Borg–host attachment. As proteins produced in the host, they may facilitate cell–cell interaction (e.g., for lateral gene transfer).

A striking observation from prior work at the wetland site is the large diversity of different and coexisting Borgs and *Methanoperedens* species [3]. Based on correlation analyses, different Borgs associate with different *Methanoperedens* [3], implying coevolution of Borgs and hosts. Lateral gene transfer has been reported as a driver for metabolic flexibility in members of the *Methanoperedenaceae* [47]. Gene acquisition (lateral gene transfer) is a prominent capacity of Borgs, which gave rise to their name and may contribute to *Methanoperedens* speciation. TR regions also may be key for establishing new Borg–host associations, especially if the aaTRs and noncoding TRs enable functional cooperation between Borg and host protein inventories. Thus, while CRISPR repeats evolve rapidly to defend against phage infection, rapid evolution of Borg TRs may be required to maintain coexistence with their hosts during coevolution.

## 4. Conclusions

We conclude that the nucleotide regions flanking repeats, and the uniqueness of the TRs in each locus, likely indicate that TRs arise from local sequences rather than being introduced from external templates. Other constraints on the mechanism behind TR formation are their generally perfect sequence repetition and A-enriched composition. Many TRs lead to aaTRs in proteins, and these are usually IDRs that are also predicted posttranslational modification sites and protein or nucleic acid binding sites. We propose that aaTR-proteins expand and modify the cellular and metabolic capacity of Borg-bearing *Methanoperedens*, yet expression of their large gene inventories is likely under tight control. Introduction of TRs in both genes and intergenic regions may be central to regulating Borg gene expression, translation, protein localization, and function. TR regions change rapidly in number and distribution to generate within population heterogeneity and between population diversity. This feature is likely central for Borg infection, association, and cospeciation of Borgs and their *Methanoperedens* hosts.

## 5. Methods

### 5.1. Identification of Borg genomes and manual genome curation

Metagenomic datasets on ggKbase (ggkbase.berkeley.edu) were searched for contigs with a dominant taxonomic profile matching *Methanoperedens* (Archaea; Euryarchaeaota; Methanomicrobia; Methanosarcinales; Candidatus Methanoperedens; Candidatus Methanoperedens nitroreducens). Manual genome binning was performed based on contig taxonomy, coverage, GC content (25% to 35% GC), and presence of nucleotide TRs.

Manual curation of Borg genomes was performed in Geneious Prime 2021.2.2 (https://www.geneious.com). It involved piecing together and verifying by paired reads placement, fragments of approximately the same GC content, sequencing reads coverage, phylogenetic profile, and relatedness to known Borgs into a single chromosome. Subsequently, careful visualization of the patterns of read discrepancies was used to locate local assembly errors, most of which were fixed by either relocating paired reads or introducing previously unplaced paired reads.

### 5.2. Genome visualization and alignments

Genomes were visualized in Geneious Prime 2021.2.2 and aligned with the MCM algorithm, or the progressiveMauve algorithm when aligning multiple contigs. Genetic neighborhood comparisons were performed using clinker [48] (v0.0.21).

### 5.3. GC skew analysis

Replichores were predicted by calculating the GC skew (G − C/G + C) and cumulative GC skew using the iRep package (gc_skew.py) [49].

### 5.4. SNP analysis

To detect SNPs in Borg genomes, we mapped reads to the individual Borg genomes using bowtie2 [50] (v.2.3.5.1, default settings), extracted mapped reads with SAMtools [51] (v.1.12), and BBMap [52] (v.38.79). The reads were remapped to the Borg genomes allowing 3% mismatch using BBMap (minid = 0.97 ambiguous = random). To detect SNPs in the mapped reads files, they were analysed with inStrain profile [11] (v.1.3.4, standard settings).

### 5.5. Tandem repeat identification

Nucleotide TRs were initially predicted in Geneious Prime 2021.2.2 (Repeat Finder, minimum repeat length 50, maximum mismatches 0) and then marked down in the completed genomes using a custom Python script (https://github.com/rohansachdeva/tandem_repeats) based on MUMmer [53] (v3.23). Nucleotide TRs were searched using a stringent threshold of ≥50 nt region and ≥3 TR units and no mismatch (—min_region_len 50—min_repeat_count 3). aaTRs were searched using a stringent threshold of ≥16 aa and ≥3 TR (-l 3—min_repeat_count 3—min_uniq_nt 1—min_region_len 16).

### 5.6. Statistical analysis

To assess the significance of the observed compositional bias between the nucleotide TRs and non-TR sequences, we performed a Kruskal–Wallis test [12]. This included separating DNA sequences into repeat and non-repeat segments and dividing these into 50 bp substrings (resulting in a total of 803 substrings from repeat DNA and 9,430 from non-repeat DNA). The incidences of each nucleotide were counted on each substring, and the values were placed into 4-member nucleotide frequency vectors [N_A_, N_T_, N_C_, N_G_]. Nucleotide frequency vectors for each 50 bp substring were then grouped into repeat and non-repeat categories and used as input to the Kruskal–Wallis test, which was implemented in SciPy (v. 1.9.0). The values were then further corrected by performing an FDR correction according to Benjamini–Yekutieli [13].

### 5.7. Secondary structure prediction

The secondary structure of TRs was predicted with RNAfold WebServer [14].

### 5.8. Protein family clustering

A dataset of 11,995 Borg proteins was constructed using all proteins from the ten curated Borg genomes and 37 additional aaTR-proteins from curated contigs of Pink, Blue, Steel, Olive, Grey, and Apricot Borg. All proteins were clustered using the fast and sensitive protein sequence searching software MMseqs2 in an all-versus-all search using sensitivity 7.5, cover 0.5, e-value 0.001 [18] (v7e2840992948ee89dcc336522dc98a74fe0adf00). The sequences of each member within a protein subfamily were aligned using result2msa of MMseqs2 and used as input to construct HMMs for each subfamily using HHblits [19]. The HMMs were then profiled against the PFAM database by HMM-HMM comparison using HHsearch [20], and protein subfamilies enriched in plasmid proteins were determined as described previously [54].

### 5.9. Functional prediction of aaTR-proteins

aaTR-proteins were profiled using InterProScan [55] (v5.51–85.0) to get functional and structural annotations of individual proteins. Protein subfamilies were functionally annotated using HMMER (hmmer.org) (v3.3, hmmsearch) and the PFAM (—cut_nc) HMM database [56]. Homology search was performed with blastp [57]. Homologous proteins were defined as members of the same protein subfamily (for Borg proteins), or best hits from blastp on NCBI or ggKbase for non-Borg proteins. Intrinsic disorder was predicted with MobiDBLite [27] and IUPred3 [28]; TMHs and cellular localization were predicted with TMHMM [58] (v2.0) and psort [59] (v2.0, archaeal mode). SLiMs were predicted with the ELM resource for functional sites in proteins [22] (http://elm.eu.org/). The single aaTR unit was queried (cell compartment: not specified), and the functional site covering most of the aaTR was usually selected (**S7 Table**). Functions of aaTR regions in proteins were also predicted with flDPnn [60].

### 5.10. Hierarchical clustering of aaTRs and construction of a phylogenetic tree

Hierarchical clustering of aaTRs was performed using triple TR units for the alignments, since three was the minimum repeat unit length set as threshold, and the regions were dynamic. The alignments were performed with MAFFT [61] (v7.453) (—treeout—reorder—localpair). The aaTR clusters were visualized and decorated in iTOL [62]. An aaTR cluster was formed when a branch contained 3≥ related sequences. The names of the clusters were given based on the most abundant amino acids found in the repeat units (amino acids represented by 10%≥ became name-giving).

The Borg DNA polymerase sequences were aligned with a reference dataset from [16], aligned with MAFFT [61] (v7.453) (—reorder—auto), trimmed with trimal [63] (v1.4.rev15) (-gt 0.2), and a maximum-likelihood tree was calculated in IQ-TREE [64] (v1.6.12) (-m TEST -st AA -bb 1000 -nt AUTO -ntmax 20 -pre). The phylogenetic tree of the DNA polymerases was visualized and decorated in iTOL [62] (v6.6).

### 5.11. Structural modeling

Structural modeling of aaTR-bearing Sm proteins was initially performed using SWISS-MODEL [65] and the best hit in the Swiss-Prot database as template. Further structural modeling of aaTR-bearing Sm proteins was performed with AlphaFold2 using ColabFold [66] and selecting the experimental option homooligomer 6. Structural modeling of aaTR-MHC proteins was performed using AlphaFold2 [67] via a LocalColabFold (—use_ptm—use_turbo—num_relax Top5—max_recycle 3) [68,69]. Modeled protein structures were visualized and superimposed onto PDB structures using PyMOL [70] (v2.3.4).

## Supporting information

S1 Fig Borg genome replication is initiated at the termini. Shown is the GC skew (grey) and cumulative GC skew (green lines). Borg DNA is replicated from the terminal inverted repeats (origin, red lines) until the terminus (terminus, blue lines).
(TIF)

S2 Fig Reads mapped to a de novo assembly showing different combinations of repeat units in a Borg population. There are two types of repeat units shown as magenta or green segments. They are identical, except for a SNP highlighted in red.
(TIF)

S3 Fig Phylogenetic placement of Borg DNA polymerases. Amino acid sequences of DNA polymerases cluster together in the B9 clade. Additional DNA polymerases present in some Borgs cluster together in the B2 clade. Reference sequences originate from Kazlauskas and colleagues [16]. The tree was rooted between the G3 and B9 clade. The data underlying this Figure can be found in Zenodo (10.5281/zenodo.6533809).
(TIF)

S4 Fig Tandem repeat unit lengths of all 460 TR regions across 10 Borgs. The data underlying this Figure can be found in **S14 Table**.
(TIF)

S5 Fig Positional nucleotide frequency and overall codon frequency in aaTR regions and non-aaTR regions of ORFs. **(A)** The positional frequency of the four nucleotides was calculated for each codon within aaTRs and all other codons. The codons were then divided into six categories (on the x-axis) based on the position of the individual nucleotides in the tripletts. One codon can fall into multiple categories. **(B)** The codon frequency in aaTR regions was divided by the codon frequency in non-aaTR regions. The same codon use would result in the value 1, codons enriched in aaTR regions have values >1, codons depleted in aaTR regions are <1, and codons absent in aaTRs are at value 0 (7 instances). The data underlying this Figure can be found in **S5 Table**.
(TIF)

S6 Fig aaTRs are enriched in disorder-promoting amino acids. The aa abundance reflects the frequency of amino acids in aaTRs divided by the frequency in all proteins. The data underlying this Figure can be found in **S8 Table** and has been sorted in terms of the propensity to introduce intrinsic disorder [71].
(EPS)

S7 Fig Length and localization of aaTR and IDR regions in aaTR proteins and non-aaTR proteins. IDRs were predicted with MobiDBLite [27] (threshold: ≥15 consecutive residues). aaTR or IDR regions were divided by the full protein length to calculate the relative length. The localization of aaTR and IDRs was calculated by dividing the mean coordinate for each region by the full sequence length. A total of 178 Borg aaTR-proteins had 220 aaTR regions (blue), and 112/178 aaTR-proteins had IDRs (green). A total of 557 Borg non-aaTR proteins had an IDR (yellow). The data underlying this Figure can be found in **S9 Table**.
(TIF)

S8 Fig Nucleotide alignment of single TR units that encode similar but distinct aaTRs. The sequences shown correspond to the data shown in **Fig 6B**.
(EPS)

S9 Fig Sequence alignment, aaTR composition, and predicted structures of Borg Sm ribonucleoproteins. (**A)** Multiple sequence alignment of Borg Sm ribonucleoproteins with and without aaTRs, Sm from *Methanoperedens* bins co-occurring with Borgs, and reference sequences from *M*. *jannaschii* (PDB: 4X9D), *M*. *nitroreducens* (WP_096203417), and *E*. *coli* (PDB: 1HK9). (**B)** aaTR units of Sm ribonucleoproteins. (**C)** Predicted structures of aaTR-Sm ribonucleoproteins.
(TIF)

S1 Table GC skew and cumulative GC skew analyses of Borg genomes. Table accompanies Figs 2 and S1. GC skew was calculated using the iRep package (Brown and colleagues, 2016) [1].
(XLSX)

S2 Table Single nucleotide variation analyses of TR regions in Borg genomes. Analysis was performed using inStrain (Olm and colleagues, 2021) [2]. SNV, single nucleotide variant, which is equivalent to SNP.
(XLSX)

S3 Table GC/AT-symmetry in regions containing TR units. Table accompanies Fig 4. The GC content was calculated in Geneious. Detailed information on each TR region can be found in S14 Table.
(XLSX)

S4 Table Nucleotide composition of Borg genomes and TRs (in %). The nucleotide composition was calculated for the coding strand of each replichore. Subsequently, a sum of both values was formed and divided by the genome length and multiplied by 100%.
(XLSX)

S5 Table A. Positional nucleotide frequency in aaTR regions and non-aaTR regions of ORFs. Table accompanying S5A Fig. NR, non-aaTR region.
(XLSX)

S6 Table Borg proteins with aaTRs. aaTR-proteins were identified using custom code available on GitHub (https://github.com/rohansachdeva/tandem_repeats).
(XLSX)

S7 Table Proteins with similar aaTR composition are part of the same repeat cluster. This Table accompanies Fig 5B. Repeat units were hierarchically clustered based on triple aaTR units. Clusters with ≥3 aaTR members were named by amino acids that form the aaTR unit (≥10% abundance, descending order). Features of select aaTRs were predicted using the eukaryotic linear motif (ELM) resource for functional sites in proteins (http://elm.eu.org/).
(XLSX)

S8 Table Relative amino acid abundance in aaTRs versus all proteins. This Table accompanies Fig 5A.
(XLSX)

S9 Table Statistics on aaTR and IDR regions in aaTR proteins and non-aaTR proteins. This Table accompanies S7 Fig.
(XLSX)

S10 Table Borg proteins with multiple aaTR regions. (XLSX)

S11 Table Small proteins with aaTRs. (XLSX)

S12 Table Sm ribonucleoproteins in Borg genomes. Features of aaTRs were predicted using the eukaryotic linear motif (ELM) resource for functional sites in proteins (http://elm.eu.org/).
(XLSX)

S13 Table Multiheme cytochromes in Borg genomes. Features of aaTRs were predicted using the eukaryotic linear motif (ELM) resource for functional sites in proteins (http://elm.eu.org/).
(XLSX)

S14 Table Statistics on TR regions in each Borg genome. TR regions were identified using custom code available on GitHub (https://github.com/rohansachdeva/tandem_repeats).
(XLSX)

S15 Table Statistics on TR regions per Borg replichore. TR regions were identified using custom code available on GitHub (https://github.com/rohansachdeva/tandem_repeats).
(XLSX)

## Abbreviations

aaTR: amino acid TR
ANME: anaerobic methanotrophic
AOM: anaerobic oxidation of methane
arcRNA: architectural RNA
ECE: extrachromosomal element
ELM: eukaryotic linear motif
FDR: false discovery rate
IDR: intrinsically disordered region
(l)ncRNA: (long) noncoding RNA
MHC: multiheme cytochrome
NCLDV: nucleocytoplasmic large DNA virus
ORF: open reading frame
PAM: protospacer adjacent motif
SLiM: short linear motif
SNP: single nucleotide polymorphism
TMH: transmembrane helix
TR: tandem repeat
VNTR: variable number TR

## References

[1] Lai S, Jia L, Subramanian B, Pan S, Zhang J, Dong Y, et al. mMGE: a database for human metagenomic extrachromosomal mobile genetic elements. Nucleic Acids Res. 2021;49:D783–D791. doi: 10.1093/nar/gkaa869 33074335 PMC7778953

[2] Yu MK, Fogarty EC, Murat Eren A. The genetic and ecological landscape of plasmids in the human gut. bioRxiv. 2022. p. 2020.11.01.361691. doi: 10.1101/2020.11.01.361691

[3] Al-Shayeb B, Schoelmerich MC, West-Roberts J, Valentin-Alvarado LE, Sachdeva R, Mullen S, et al. Borgs are giant genetic elements with potential to expand metabolic capacity. Nature. 2022. doi: 10.1038/s41586-022-05256-1 36261517 PMC9605863

[4] Schoelmerich MC, Ouboter HT, Sachdeva R, Penev PI, Amano Y, West-Roberts J, et al. A widespread group of large plasmids in methanotrophic Methanoperedens archaea. Nat Commun. 2022;13:1–11. 36400771 10.1038/s41467-022-34588-9 PMC9674854

[5] Haroon MF, Hu S, Shi Y, Imelfort M, Keller J, Hugenholtz P, et al. Anaerobic oxidation of methane coupled to nitrate reduction in a novel archaeal lineage. Nature. 2013;500:567–570. doi: 10.1038/nature12375 23892779

[6] Koonin EV, Yutin N. Evolution of the Large Nucleocytoplasmic DNA Viruses of Eukaryotes and Convergent Origins of Viral Gigantism. Adv Virus Res. 2019;103:167–202. doi: 10.1016/bs.aivir.2018.09.002 30635076

[7] Wang H, Peng N, Shah SA, Huang L, She Q. Archaeal extrachromosomal genetic elements. Microbiol Mol Biol Rev. 2015;79:117–152. doi: 10.1128/MMBR.00042-14 25694123 PMC4402960

[8] Gunge N, Fukuda K, Takahashi S, Meinhardt F. Migration of the yeast linear DNA plasmid from the cytoplasm into the nucleus in Saccharomyces cerevisiae. Curr Genet. 1995;28:280–288. doi: 10.1007/BF00309788 8529275

[9] Chater KF, Kinashi H. Streptomyces Linear Plasmids: Their Discovery,Functions, Interactions with Other Replicons, and Evolutionary Significance. In: Meinhardt F, Klassen R, editors. Microbial Linear Plasmids. Berlin, Heidelberg: Springer Berlin Heidelberg; 2007. pp. 1–31.

[10] Wagenknecht M, Dib JR, Thürmer A, Daniel R, Farías ME, Meinhardt F. Structural peculiarities of linear megaplasmid, pLMA1, from Micrococcus luteus interfere with pyrosequencing reads assembly. Biotechnol Lett. 2010;32:1853–1862. doi: 10.1007/s10529-010-0357-y 20652620 PMC2974207

[11] Olm MR, Crits-Christoph A, Bouma-Gregson K, Firek BA, Morowitz MJ, Banfield JF. inStrain profiles population microdiversity from metagenomic data and sensitively detects shared microbial strains. Nat Biotechnol. 2021;39:727–736. doi: 10.1038/s41587-020-00797-0 33462508 PMC9223867

[12] Kruskal WH, Wallis WA. Use of Ranks in One-Criterion Variance Analysis. J Am Stat Assoc. 1952;47:583–621.

[13] Benjamini Y, Yekutieli D. The control of the false discovery rate in multiple testing under dependency. aos 2001;29:1165–1188.

[14] Gruber AR, Lorenz R, Bernhart SH, Neuböck R, Hofacker IL. The Vienna RNA websuite. Nucleic Acids Res. 2008;36:W70–W74. doi: 10.1093/nar/gkn188 18424795 PMC2447809

[15] Kim JC, Mirkin SM. The balancing act of DNA repeat expansions. Curr Opin Genet Dev. 2013;23:280–288. doi: 10.1016/j.gde.2013.04.009 23725800 PMC3703482

[16] Kazlauskas D, Krupovic M, Guglielmini J, Forterre P, Venclovas Č. Diversity and evolution of B-family DNA polymerases. Nucleic Acids Res. 2020;48:10142–10156. doi: 10.1093/nar/gkaa760 32976577 PMC7544198

[17] van der Lee R, Buljan M, Lang B, Weatheritt RJ, Daughdrill GW, Dunker AK, et al. Classification of intrinsically disordered regions and proteins. Chem Rev. 2014;114:6589–6631. doi: 10.1021/cr400525m 24773235 PMC4095912

[18] Hauser M, Steinegger M, Söding J. MMseqs software suite for fast and deep clustering and searching of large protein sequence sets. Bioinformatics. 2016;32:1323–1330. doi: 10.1093/bioinformatics/btw006 26743509

[19] Remmert M, Biegert A, Hauser A, Söding J. HHblits: lightning-fast iterative protein sequence searching by HMM-HMM alignment. Nat Methods. 2011;9:173–175. doi: 10.1038/nmeth.1818 22198341

[20] Söding J. Protein homology detection by HMM-HMM comparison. Bioinformatics. 2005;21:951–60. doi: 10.1093/bioinformatics/bti125 15531603

[21] Van Roey K, Uyar B, Weatheritt RJ, Dinkel H, Seiler M, Budd A, et al. Short linear motifs: ubiquitous and functionally diverse protein interaction modules directing cell regulation. Chem Rev. 2014;114:6733–78. doi: 10.1021/cr400585q 24926813

[22] Kumar M, Gouw M, Michael S, Sámano-Sánchez H, Pancsa R, Glavina J, et al. ELM-the eukaryotic linear motif resource in 2020. Nucleic Acids Res. 2020;48:D296–D306. doi: 10.1093/nar/gkz1030 31680160 PMC7145657

[23] Maupin-Furlow J. Proteasomes and protein conjugation across domains of life. Nat Rev Microbiol. 2011;10:100–111. doi: 10.1038/nrmicro2696 22183254 PMC3291102

[24] Garnham CP, Roll-Mecak A. The chemical complexity of cellular microtubules: tubulin post-translational modification enzymes and their roles in tuning microtubule functions. Cytoskeleton. 2012;69:442–63. doi: 10.1002/cm.21027 22422711 PMC3459347

[25] Vogel J, Luisi BF. Hfq and its constellation of RNA. Nat Rev Microbiol. 2011;9:578–89. doi: 10.1038/nrmicro2615 21760622 PMC4615618

[26] Nikulin A, Mikhailina A, Lekontseva N, Balobanov V, Nikonova E, Tishchenko S. Characterization of RNA-binding properties of the archaeal Hfq-like protein from Methanococcus jannaschii. J Biomol Struct Dyn. 2017;35:1615–28. doi: 10.1080/07391102.2016.1189849 27187760

[27] Necci M, Piovesan D, Dosztányi Z, Tosatto SCE. MobiDB-lite: fast and highly specific consensus prediction of intrinsic disorder in proteins. Bioinformatics. 2017;33:1402–4. doi: 10.1093/bioinformatics/btx015 28453683

[28] Erdős G, Pajkos M, Dosztányi Z. IUPred3: prediction of protein disorder enhanced with unambiguous experimental annotation and visualization of evolutionary conservation. Nucleic Acids Res. 2021;49:W297–W303. doi: 10.1093/nar/gkab408 34048569 PMC8262696

[29] Kletzin A, Heimerl T, Flechsler J, van Niftrik L, Rachel R, Klingl A. Cytochromes c in Archaea: distribution, maturation, cell architecture, and the special case of Ignicoccus hospitalis. Front Microbiol. 2015;6:439. doi: 10.3389/fmicb.2015.00439 26029183 PMC4429474

[30] Ryan CP. Tandem repeat disorders. Evol Med Public Health. 2019;2019:17. doi: 10.1093/emph/eoz005 30800316 PMC6379701

[31] Usdin K. The biological effects of simple tandem repeats: lessons from the repeat expansion diseases. Genome Res. 2008;18:1011–19. doi: 10.1101/gr.070409.107 18593815 PMC3960014

[32] Fondon JW 3rd, Garner HR. Molecular origins of rapid and continuous morphological evolution. Proc Natl Acad Sci U S A. 2004;101:18058–18063. doi: 10.1073/pnas.0408118101 15596718 PMC539791

[33] Viguera E, Canceill D, Ehrlich SD. Replication slippage involves DNA polymerase pausing and dissociation. EMBO J. 2001;20:2587–95. doi: 10.1093/emboj/20.10.2587 11350948 PMC125466

[34] Zhou K, Aertsen A, Michiels CW. The role of variable DNA tandem repeats in bacterial adaptation. FEMS Microbiol Rev. 2014;38:119–141. doi: 10.1111/1574-6976.12036 23927439

[35] Castillo-Lizardo M, Henneke G, Viguera E. Replication slippage of the thermophilic DNA polymerases B and D from the Euryarchaeota Pyrococcus abyssi. Front Microbiol. 2014;5:403. doi: 10.3389/fmicb.2014.00403 25177316 PMC4134008

[36] Tyson GW, Banfield JF. Rapidly evolving CRISPRs implicated in acquired resistance of microorganisms to viruses. Environ Microbiol. 2008;10:200–7. doi: 10.1111/j.1462-2920.2007.01444.x 17894817

[37] McGinn J, Marraffini LA. Molecular mechanisms of CRISPR-Cas spacer acquisition. Nat Rev Microbiol. 2019;17:7–12. doi: 10.1038/s41579-018-0071-7 30171202

[38] Waters TR, Swann PF. Thymine-DNA glycosylase and G to A transition mutations at CpG sites. Mutat Res. 2000;462:137–147. doi: 10.1016/s1383-5742(00)00031-4 10767625

[39] Statello L, Guo C-J, Chen L-L, Huarte M. Gene regulation by long non-coding RNAs and its biological functions. Nat Rev Mol Cell Biol. 2021;22:96–118. doi: 10.1038/s41580-020-00315-9 33353982 PMC7754182

[40] Ninomiya K, Hirose T. Short Tandem Repeat-Enriched Architectural RNAs in Nuclear Bodies: Functions and Associated Diseases. Noncoding. RNA. 2020:6. doi: 10.3390/ncrna6010006 32093161 PMC7151548

[41] Coelho Ribeiro M de L, Espinosa J, Islam S, Martinez O, Thanki JJ, Mazariegos S, et al. Malleable ribonucleoprotein machine: protein intrinsic disorder in the Saccharomyces cerevisiae spliceosome. PeerJ. 2013;1:e2. doi: 10.7717/peerj.2 23638354 PMC3628832

[42] Törö I, Thore S, Mayer C, Basquin J, Séraphin B, Suck D. RNA binding in an Sm core domain: X-ray structure and functional analysis of an archaeal Sm protein complex. EMBO J. 2001;20:2293–2303. doi: 10.1093/emboj/20.9.2293 11331594 PMC125243

[43] Haynes C, Oldfield CJ, Ji F, Klitgord N, Cusick ME, Radivojac P, et al. Intrinsic disorder is a common feature of hub proteins from four eukaryotic interactomes. PLoS Comput Biol. 2006;2:e100. doi: 10.1371/journal.pcbi.0020100 16884331 PMC1526461

[44] Breuer M, Rosso KM, Blumberger J. Electron flow in multiheme bacterial cytochromes is a balancing act between heme electronic interaction and redox potentials. Proc Natl Acad Sci U S A. 2014;111:611–616. doi: 10.1073/pnas.1316156111 24385579 PMC3896160

[45] Yakhnina AA, Bernhardt TG. The Tol-Pal system is required for peptidoglycan-cleaving enzymes to complete bacterial cell division. Proc Natl Acad Sci U S A. 2020;117:6777–6783. doi: 10.1073/pnas.1919267117 32152098 PMC7104345

[46] Heilpern AJ, Waldor MK. CTXphi infection of Vibrio cholerae requires the tolQRA gene products. J Bacteriol. 2000;182:1739–1747. doi: 10.1128/JB.182.6.1739-1747.2000 10692381 PMC94473

[47] Leu AO, McIlroy SJ, Ye J, Parks DH, Orphan VJ, Tyson GW. Lateral Gene Transfer Drives Metabolic Flexibility in the Anaerobic Methane-Oxidizing Archaeal Family Methanoperedenaceae. MBio. 2020:11. doi: 10.1128/mBio.01325-20 32605988 PMC7327174

[48] Gilchrist CLM, Chooi Y-H. Clinker & clustermap.js: Automatic generation of gene cluster comparison figures. Bioinformatics. 2021. doi: 10.1093/bioinformatics/btab007 33459763

[49] Brown CT, Olm MR, Thomas BC, Banfield JF. Measurement of bacterial replication rates in microbial communities. Nat Biotechnol. 2016;34:1256–1263. doi: 10.1038/nbt.3704 27819664 PMC5538567

[50] Langmead B, Salzberg SL. Fast gapped-read alignment with Bowtie 2. Nat Methods. 2012;9:357–359. doi: 10.1038/nmeth.1923 22388286 PMC3322381

[51] Li H, Handsaker B, Wysoker A, Fennell T, Ruan J, Homer N, et al. The Sequence Alignment/Map format and SAMtools. Bioinformatics. 2009:2078–2079. doi: 10.1093/bioinformatics/btp352 19505943 PMC2723002

[52] Bushnell B. BBMap: A fast, accurate, splice-aware aligner. Lawrence Berkeley National Lab. (LBNL), Berkeley, CA (United States); 2014 Mar. Report No.: LBNL-7065E. Available from: https://www.osti.gov/biblio/1241166-bbmap-fast-accurate-splice-aware-aligner

[53] Kurtz S, Phillippy A, Delcher AL, Smoot M, Shumway M, Antonescu C, et al. Versatile and open software for comparing large genomes. Genome Biol. 2004;5:R12. doi: 10.1186/gb-2004-5-2-r12 14759262 PMC395750

[54] Jaffe AL, Thomas AD, He C, Keren R, Valentin-Alvarado LE, Munk P, et al. Patterns of Gene Content and Co-occurrence Constrain the Evolutionary Path toward Animal Association in Candidate Phyla Radiation Bacteria. MBio. 2021;12:e0052121. doi: 10.1128/mBio.00521-21 34253055 PMC8406219

[55] Jones P, Binns D, Chang H-Y, Fraser M, Li W, McAnulla C, et al. InterProScan 5: genome-scale protein function classification. Bioinformatics. 2014;30:1236–1240. doi: 10.1093/bioinformatics/btu031 24451626 PMC3998142

[56] Finn RD, Bateman A, Clements J, Coggill P, Eberhardt RY, Eddy SR, et al. Pfam: the protein families database. Nucleic Acids Res. 2014;42:D222–D230. doi: 10.1093/nar/gkt1223 24288371 PMC3965110

[57] Altschul SF, Gish W, Miller W, Myers EW, Lipman DJ. Basic local alignment search tool. J Mol Biol. 1990;215:403–410. doi: 10.1016/S0022-2836(05)80360-2 2231712

[58] Krogh A, Larsson B, von Heijne G, Sonnhammer EL. Predicting transmembrane protein topology with a hidden Markov model: application to complete genomes. J Mol Biol. 2001;305:567–580. doi: 10.1006/jmbi.2000.4315 11152613

[59] Yu NY, Wagner JR, Laird MR, Melli G, Rey S, Lo R, et al. PSORTb 3.0: improved protein subcellular localization prediction with refined localization subcategories and predictive capabilities for all prokaryotes. Bioinformatics. 2010;26:1608–1615. doi: 10.1093/bioinformatics/btq249 20472543 PMC2887053

[60] Hu G, Katuwawala A, Wang K, Wu Z, Ghadermarzi S, Gao J, et al. flDPnn: Accurate intrinsic disorder prediction with putative propensities of disorder functions. Nat Commun. 2021;12:4438. doi: 10.1038/s41467-021-24773-7 34290238 PMC8295265

[61] Katoh K, Misawa K, Kuma K-I, Miyata T. MAFFT: a novel method for rapid multiple sequence alignment based on fast Fourier transform. Nucleic Acids Res. 2002;30:3059–3066. doi: 10.1093/nar/gkf436 12136088 PMC135756

[62] Letunic I, Bork P. Interactive tree of life (iTOL) v3: an online tool for the display and annotation of phylogenetic and other trees. Nucleic Acids Res. 2016;44:W242–W245. doi: 10.1093/nar/gkw290 27095192 PMC4987883

[63] Capella-Gutiérrez S, Silla-Martínez JM, Gabaldón T. trimAl: a tool for automated alignment trimming in large-scale phylogenetic analyses. Bioinformatics. 2009;25:1972–1973. doi: 10.1093/bioinformatics/btp348 19505945 PMC2712344

[64] Nguyen L-T, Schmidt HA, von Haeseler A, Minh BQ. IQ-TREE: a fast and effective stochastic algorithm for estimating maximum-likelihood phylogenies. Mol Biol Evol. 2015;32:268–274. doi: 10.1093/molbev/msu300 25371430 PMC4271533

[65] Waterhouse A, Bertoni M, Bienert S, Studer G, Tauriello G, Gumienny R, et al. SWISS-MODEL: homology modelling of protein structures and complexes. Nucleic Acids Res. 2018;46:W296–W303. doi: 10.1093/nar/gky427 29788355 PMC6030848

[66] Mirdita M, Ovchinnikov S, Steinegger M. ColabFold—Making protein folding accessible to all. bioRxiv. 2021. p. 2021.08.15.456425. doi: 10.1101/2021.08.15.456425 PMC9184281 35637307

[67] Jumper J, Evans R, Pritzel A, Green T, Figurnov M, Ronneberger O, et al. Highly accurate protein structure prediction with AlphaFold. Nature. 2021;596:583–589. doi: 10.1038/s41586-021-03819-2 34265844 PMC8371605

[68] Mirdita M, Schütze K, Moriwaki Y, Heo L, Ovchinnikov S, Steinegger M. ColabFold—Making protein folding accessible to all. Research Square. 2021. doi: 10.21203/rs.3.rs-1032816/v1 PMC9184281 35637307

[69] Moriwaki Y. localcolabfold: ColabFold on your local PC. Github; Available from: https://github.com/YoshitakaMo/localcolabfold

[70] Delano WL. The PyMOL Molecular Graphics System. http://www.pymol.org. 2002 [cited 2022 Jan 27]. Available from: https://ci.nii.ac.jp/naid/10020095229/

[71] Uversky VN. Intrinsically disordered proteins and their “mysterious” (meta)physics. Front Physiol. 2019:7. doi: 10.3389/fphy.2019.00010

