# Supplementary figures and images for: Tandem repeats in giant archaeal Borg elements undergo rapid evolution and create new intrinsically disordered regions in proteins
Source: PLoS Biol. 2023 Jan 26;21(1):e3001980. doi: 10.1371/journal.pbio.3001980 (PMC9879509; doi:10.1371/journal.pbio.3001980)

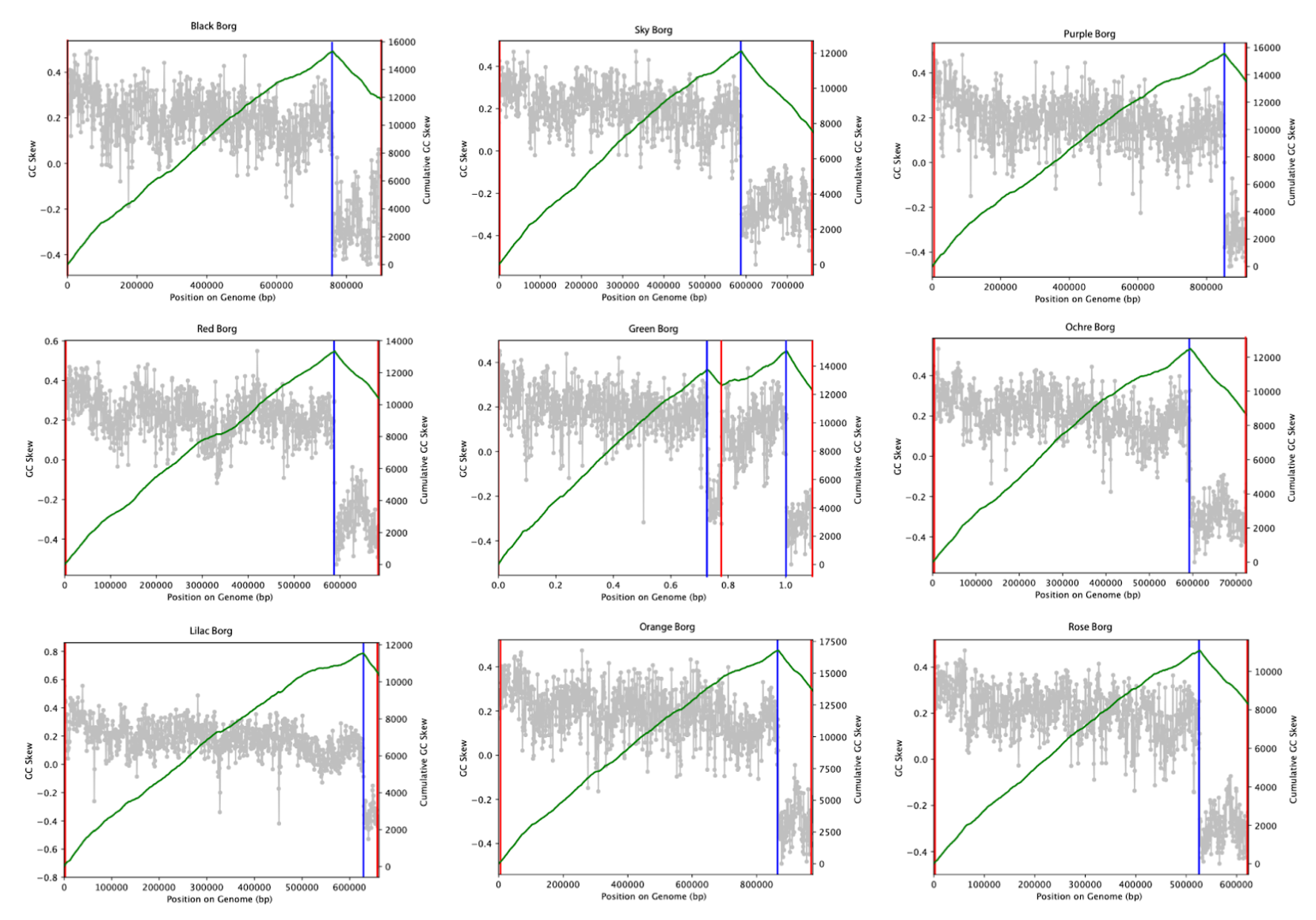

Supplement: S1 Fig — Shown is the GC skew (grey) and cumulative GC skew (green lines). Borg DNA is replicated from the terminal inverted repeats (origin, red lines) until the terminus (terminus, blue lines). (TIF) [file pbio.3001980.s001.tif]

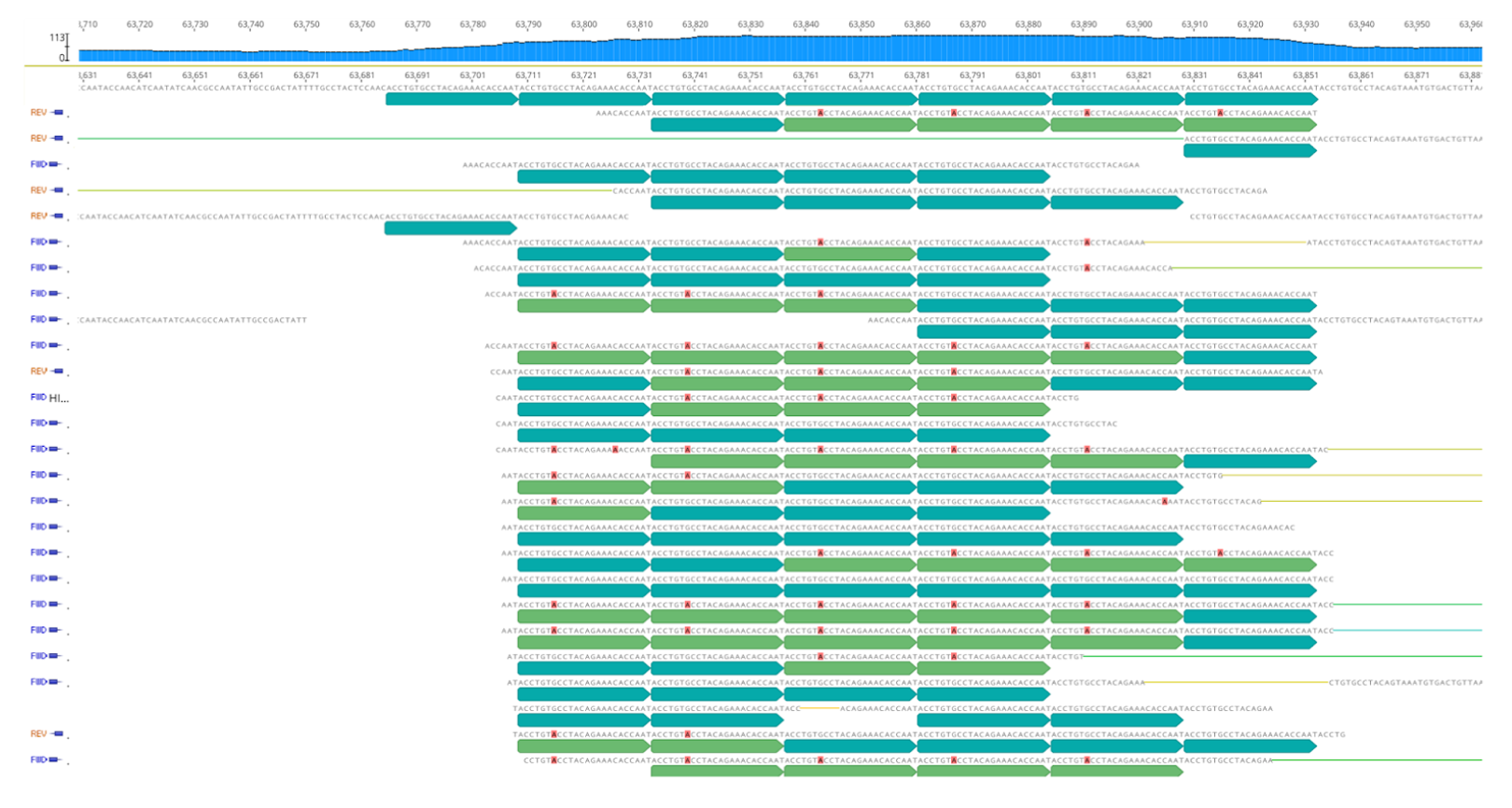

Supplement: S2 Fig — There are two types of repeat units shown as magenta or green segments. They are identical, except for a SNP highlighted in red. (TIF) [file pbio.3001980.s002.tif]

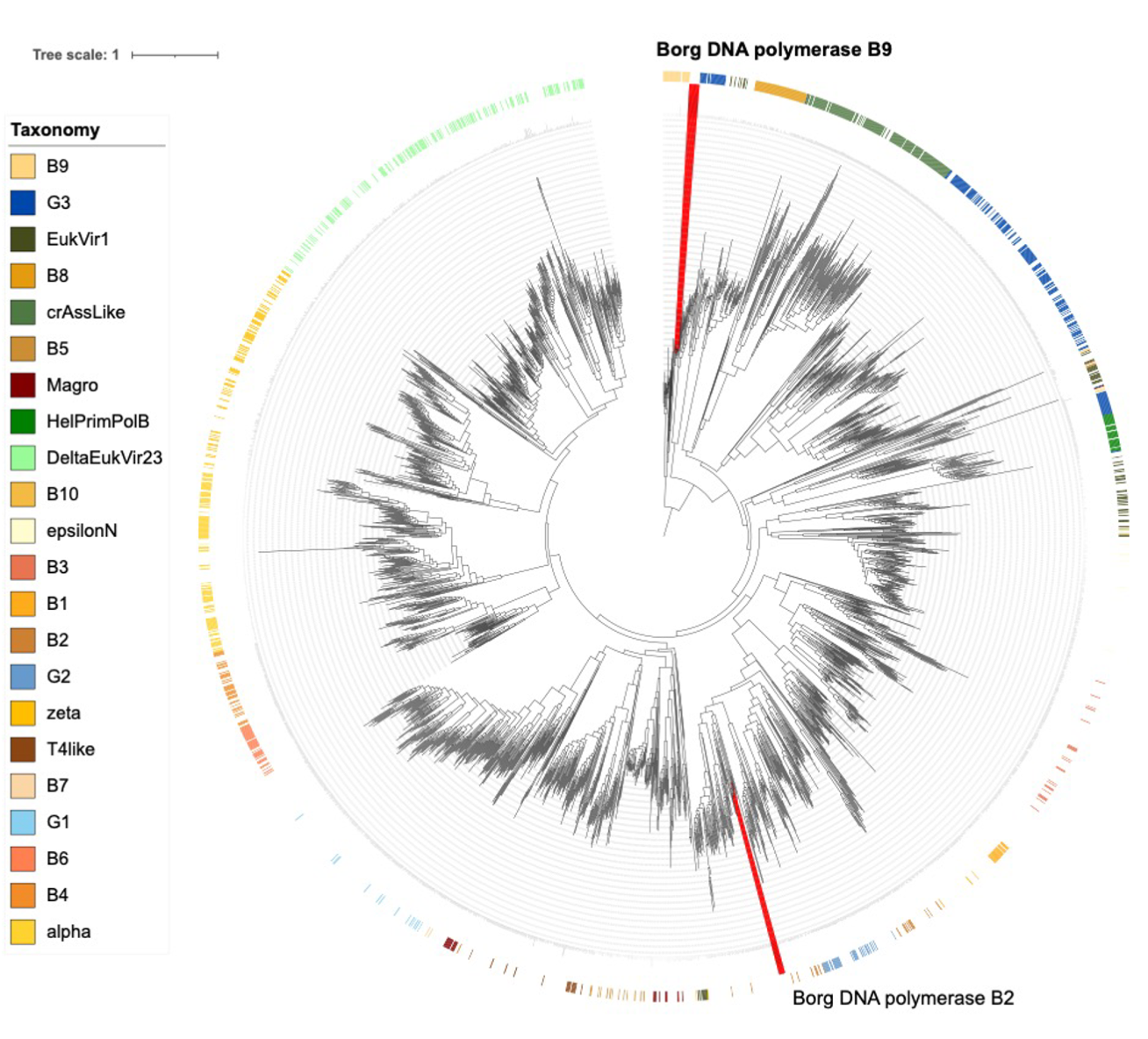

Supplement: S3 Fig — Amino acid sequences of DNA polymerases cluster together in the B9 clade. Additional DNA polymerases present in some Borgs cluster together in the B2 clade. Reference sequences originate from Kazlauskas and colleagues [16]. The tree was rooted between the G3 and B9 clade. The data underlying this Figure can be found in Zenodo (10.5281/zenodo.6533809). (TIF) [file pbio.3001980.s003.tif]

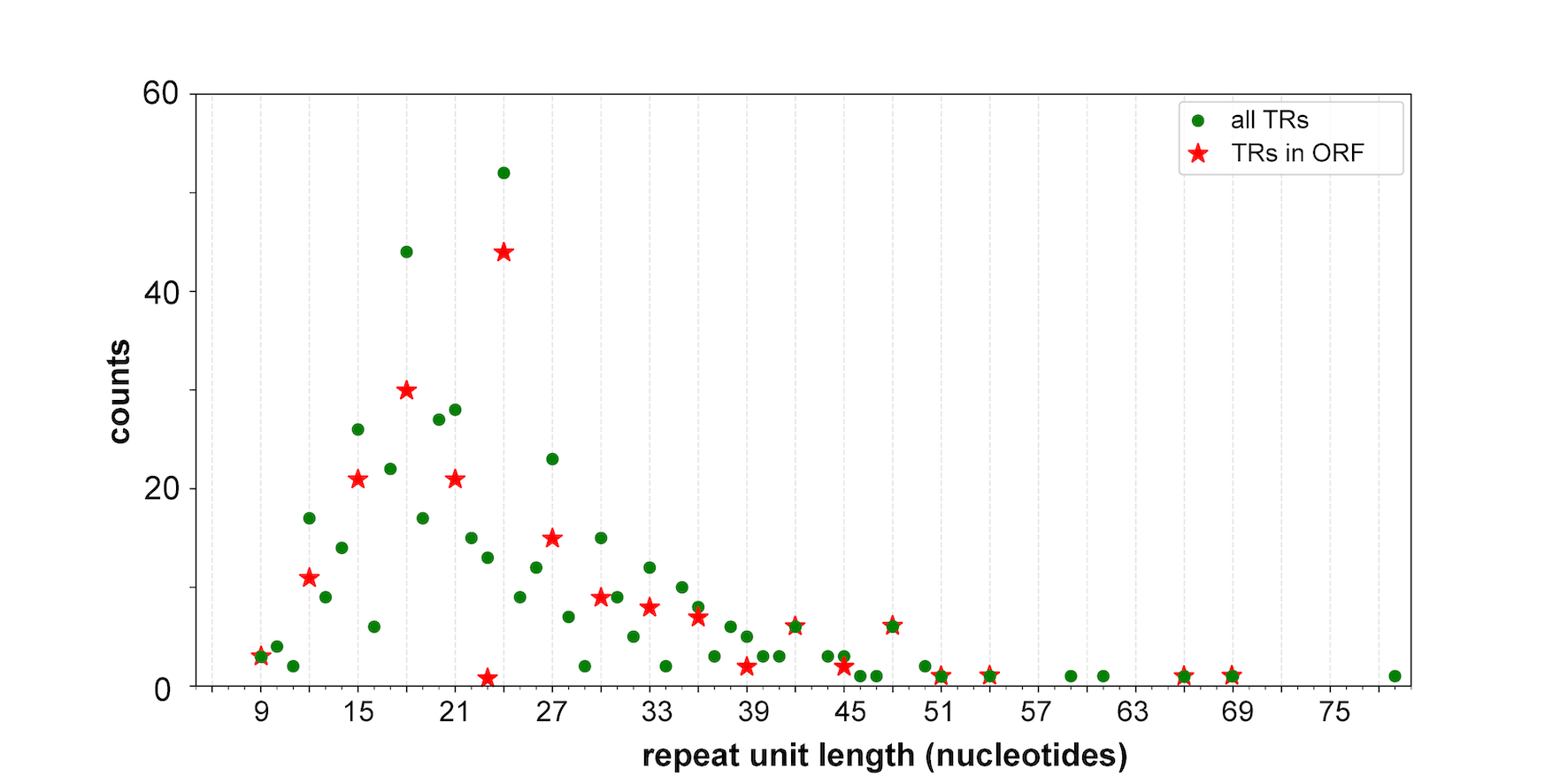

Supplement: S4 Fig — The data underlying this Figure can be found in S14 Table. (TIF) [file pbio.3001980.s004.tif]

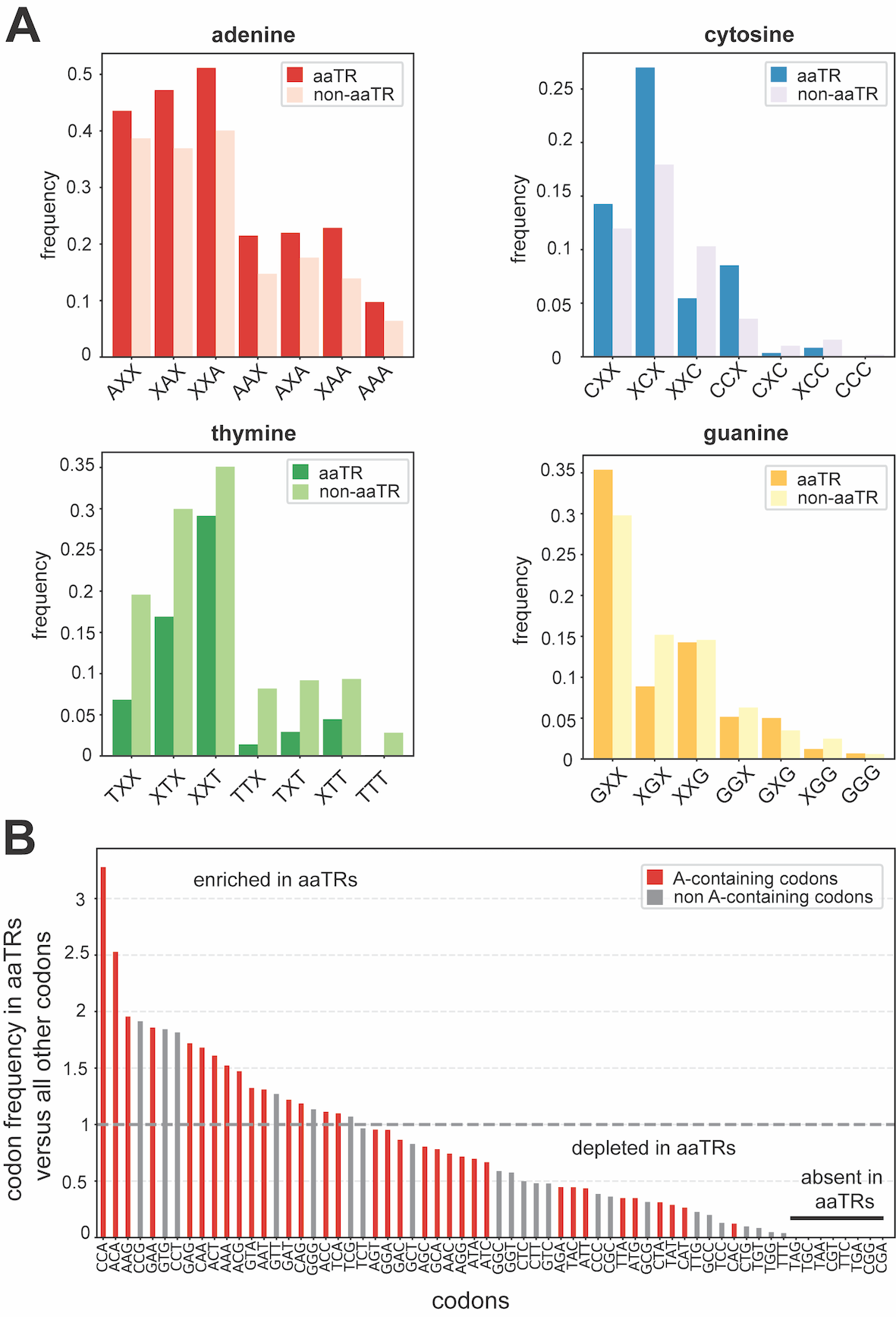

Supplement: S5 Fig — (A) The positional frequency of the four nucleotides was calculated for each codon within aaTRs and all other codons. The codons were then divided into six categories (on the x-axis) based on the position of the individual nucleotides in the tripletts. One codon can fall into multiple categories. (B) The codon frequency in aaTR regions was divided by the codon frequency in non-aaTR regions. The same codon use would result in the value 1, codons enriched in aaTR regions have values >1, codons depleted in aaTR regions are <1, and codons absent in aaTRs are at value 0 (7 instances). The data underlying this Figure can be found in S5 Table. (TIF) [file pbio.3001980.s005.tif]

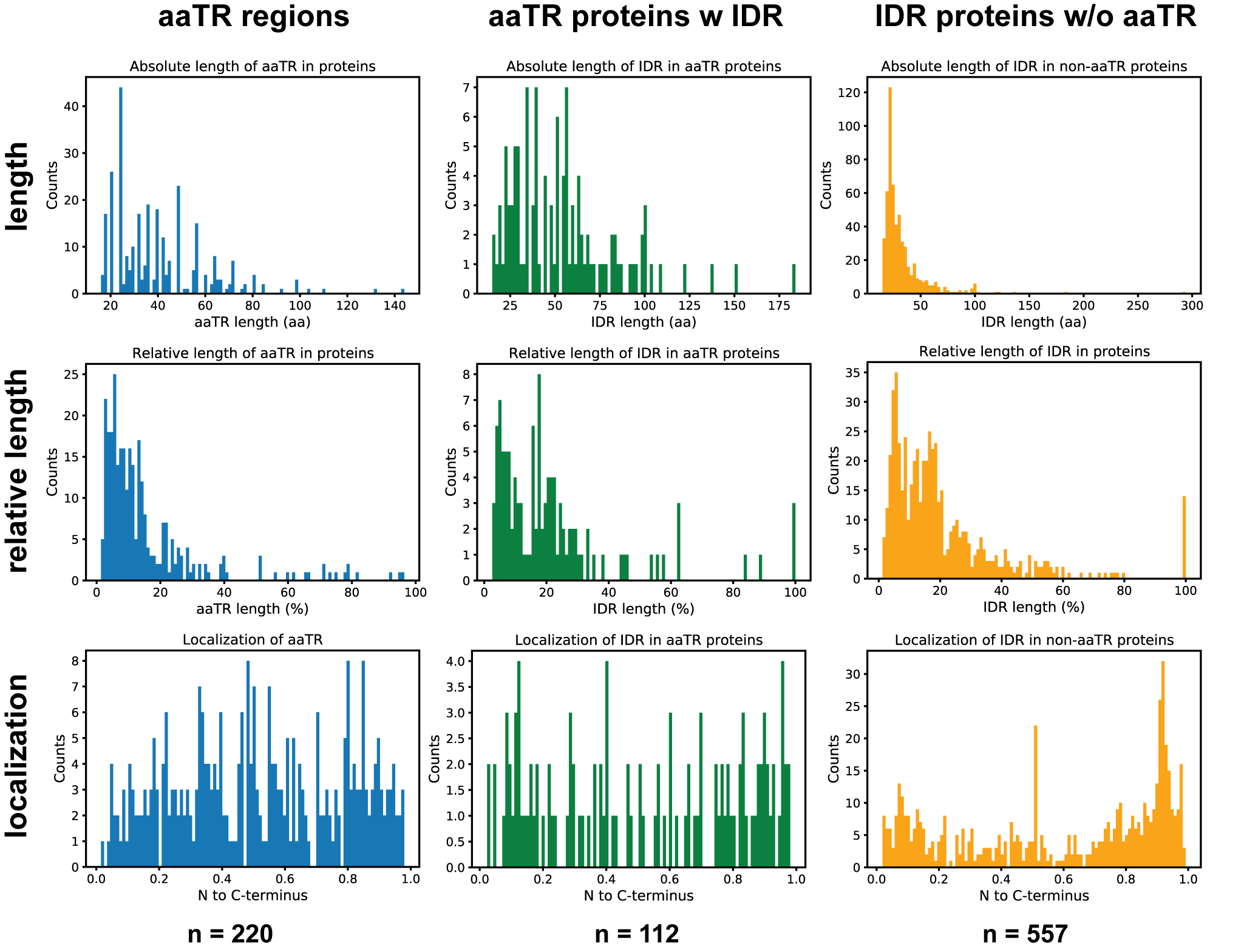

Supplement: S7 Fig — IDRs were predicted with MobiDBLite [27] (threshold: ≥15 consecutive residues). aaTR or IDR regions were divided by the full protein length to calculate the relative length. The localization of aaTR and IDRs was calculated by dividing the mean coordinate for each region by the full sequence length. A total of 178 Borg aaTR-proteins had 220 aaTR regions (blue), and 112/178 aaTR-proteins had IDRs (green). A total of 557 Borg non-aaTR proteins had an IDR (yellow). The data underlying this Figure can be found in S9 Table. (TIF) [file pbio.3001980.s007.tif]

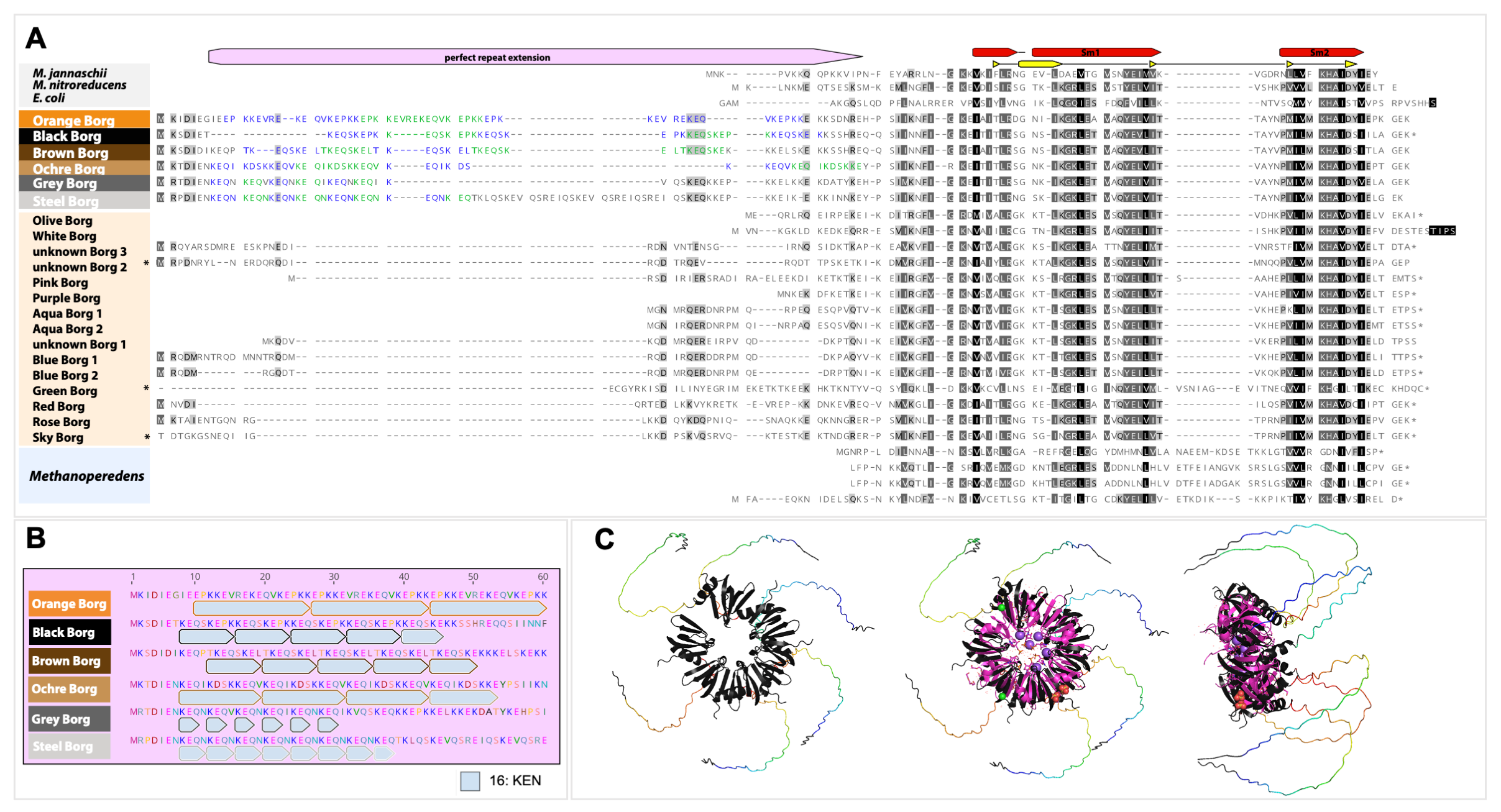

Supplement: S9 Fig — (A) Multiple sequence alignment of Borg Sm ribonucleoproteins with and without aaTRs, Sm from Methanoperedens bins co-occurring with Borgs, and reference sequences from M. jannaschii (PDB: 4X9D), M. nitroreducens (WP_096203417), and E. coli (PDB: 1HK9). (B) aaTR units of Sm ribonucleoproteins. (C) Predicted structures of aaTR-Sm ribonucleoproteins. (TIF) [file pbio.3001980.s009.tif]
